# Investigation on biomechanical responses in bilateral semicircular canals and nystagmus in vestibulo-ocular reflex experiments under different forward-leaning angles

**DOI:** 10.3389/fbioe.2024.1322008

**Published:** 2024-02-07

**Authors:** Jing Zhang, Shili Zhang, Yue Li, Lijie Xiao, Shen Yu, Xiang Wu, Shuang Shen, Hang Xu

**Affiliations:** ^1^ School of Medical Imaging, Xuzhou Medical University, Xuzhou, China; ^2^ Department of Otolaryngology, Affiliated Hospital of Xuzhou Medical University, Xuzhou, China; ^3^ Department of Neurology, General Hospital of Xuzhou Mining Group, Xuzhou, China; ^4^ State Key Laboratory of Structural Analysis for Industrial Equipment, Dalian University of Technology, Dalian, China; ^5^ School of Rehabilitation Medicine, Binzhou Medical University, Yantai, China

**Keywords:** semicircular canals, vestibulo-ocular reflex, biomechanical responses of the cupula, nystagmus, symmetry

## Abstract

Different head positions affect the responses of the vestibular semicircular canals (SCCs) to angular movement. Specific head positions can relieve vestibular disorders caused by excessive stimulating SCCs. In this study, we quantitatively explored responses of human SCCs using numerical simulations of fluid-structure interaction and vestibulo-ocular reflex (VOR) experiments under different forward-leaning angles of the head, including 0°, 10°, 20°, 30°, 40°, 50°, and 60°. It was found that the horizontal nystagmus slow-phase velocity and corresponding biomechanical responses of the cupula in horizontal SCC increased with the forward-leaning angles of the head, reached a maximum when the head was tilted 30° forward, and then gradually decreased. However, no obvious vertical or torsional nystagmus was observed in the VOR experiments. In the numerical model of bilateral SCCs, the biomechanical responses of the cupula in the left anterior SCC and the right anterior SCC showed the same trends; they decreased with the forward-leaning angles, reached a minimum at a 40° forward tilt of the head, and then gradually increased. Similarly, the biomechanical responses of the cupula in the left posterior SCC and in the right posterior SCC followed a same trend, decreasing with the forward-leaning angles, reaching a minimum at a 30° forward tilt of the head, and then gradually increasing. Additionally, the biomechanical responses of the cupula in both the anterior and posterior SCCs consistently remained lower than those observed in the horizontal SCCs across all measured head positions. The occurrence of these numerical results was attributed to the consistent maintenance of mutual symmetry in the bilateral SCCs with respect to the mid-sagittal plane containing the axis of rotation. This symmetry affected the distribution of endolymph pressure, resulting in biomechanical responses of the cupula in each pair of symmetrical SCCs exhibiting same tendencies under different forward-leaning angles of the head. These results provided a reliable numerical basis for future research to relieve vestibular diseases induced by spatial orientation of SCCs.

## 1 Introduction

The vestibular semicircular canals (SCCs) in the human inner ear can detect the angular motion of the head (Jaeger et al., 2002; Hullar and Williams, 2006; Zuma e Maia et al., 2020; Yu et al., 2021), which play an important role in maintaining body balance and visual stability. The SCCs are located in the labyrinthine cavity of the temporal bone, which includes the horizontal, anterior, and posterior SCCs. The planes of each canal are approximately orthogonal to each other. When the SCCs experience angular motion, the endolymph fluid interacts with the cupulae because of inertia, the cupulae are deflected, and the bundle of sensory hair cells embedded in the cristae are bent. The cupulae in the vestibular SCCs plays a role in sensing both velocity and acceleration (Boselli et al., 2013; Goyens et al., 2019). Based on the principle of the vestibulo-ocular reflex (VOR) (Squires et al., 2004; Boselli et al., 2014; Rabbitt, 2019; Giannoni et al., 2020; Rey-Martinez et al., 2020), involuntary eye movements called nystagmus are generated. Slow-phase velocity (SPV) is a characteristic of nystagmus, which has a quantitative relationship with maximal cupula displacements and shear strain (Wu et al., 2020).

Most angular movements of the head do not generally occur in a single corresponding SCC plane. The labyrinth usually decomposes the rotational stimulus to the head into components along the direction of the three SCCs. Each SCC undergoes excitation or inhibition, producing and transmitting neural signals to the brain, which eventually causes contraction or relaxation of the corresponding extraocular muscles to induce nystagmus. The type of nystagmus and the magnitude of SPV are determined by the effects of excitation or inhibition generated by different SCCs. Rabbitt (1999) developed a mathematical model of three SCCs in toadfish and found that the volume displacements of the cupulae obey a simple cosine rule when the tilt angles of the SCCs change. Moreover, Wu et al. (2021a) investigated the biomechanical response of human SCCs and nystagmus SPV under different left-leaning head positions. They found that a variation in head positions affected the distribution of endolymphatic fluid pressure in SCCs. In addition, these studies suggested that the cupula response in each pair of SCCs located approximately in the same plane had similar variation trends and approximately complied with the cosine law, providing a quantitative numerical basis for analysing the magnitude and type of nystagmus under different left-leaning head positions.

**FIGURE 1:**
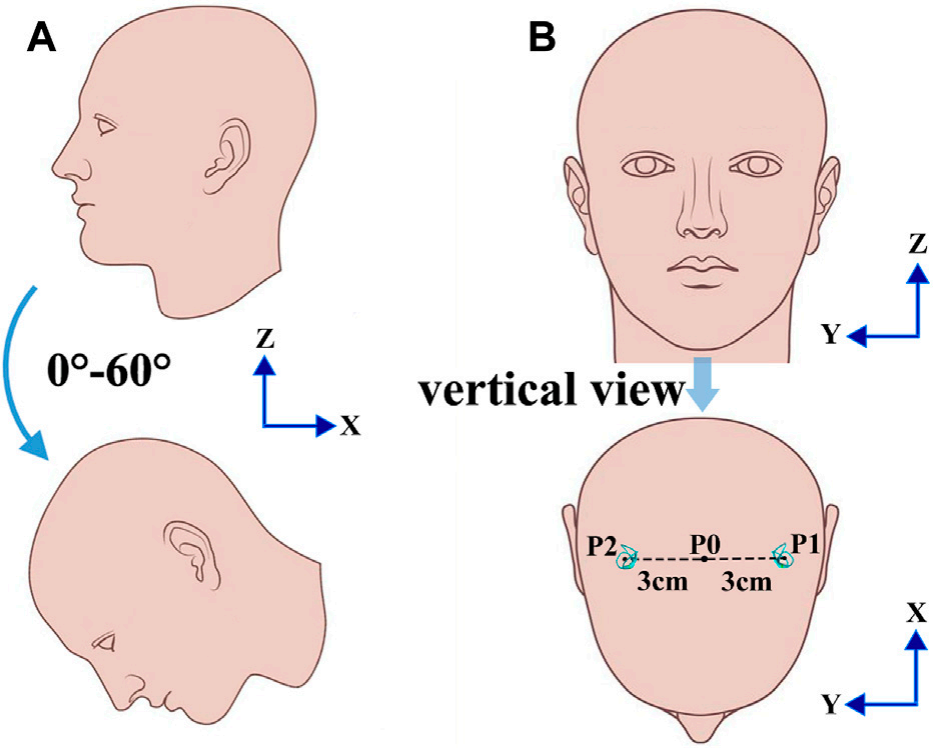
Different head positions. **(A)** Different positions of the head tilted 0°, 10°, 20°, 30°, 40°, 50°, and 60° forward. **(B)** The location of SCCs with a normal head position.

However, for any rotation axis included in the midsagittal plane of the head, the positions of bilateral SCCs are mutually symmetric. Considering the pressure gradient of the endolymph fluid in the SCCs distributed along the direction of rotation, the transcupular pressure in mutually symmetric SCCs with respect to the mid-sagittal plane containing the axis of rotation may be similar when the head experiences a rotational stimulus. These conditions markedly differ from those observed in left-leaning head positions, where significant effects on the biomechanical response of each SCC to the angular motion of the head and the functional response of the VOR may result from the combined effects of excitation or inhibition generated by each SCC. In this study, we aimed to quantitatively explore the biomechanical responses of the cupulae in each SCC and the volunteers’ nystagmus SPV under different forward-leaning angles of the head by numerical simulation of the SCC model in human and VOR experiments. The forward-leaning angles of the head we investigated included the head tilted at 0°, 10°, 20°, 30°, 40°, 50°, and 60° forward (see Figure 1A).

## 2 Materials and methods

### 2.1 Model of SCCs

**FIGURE 2:**
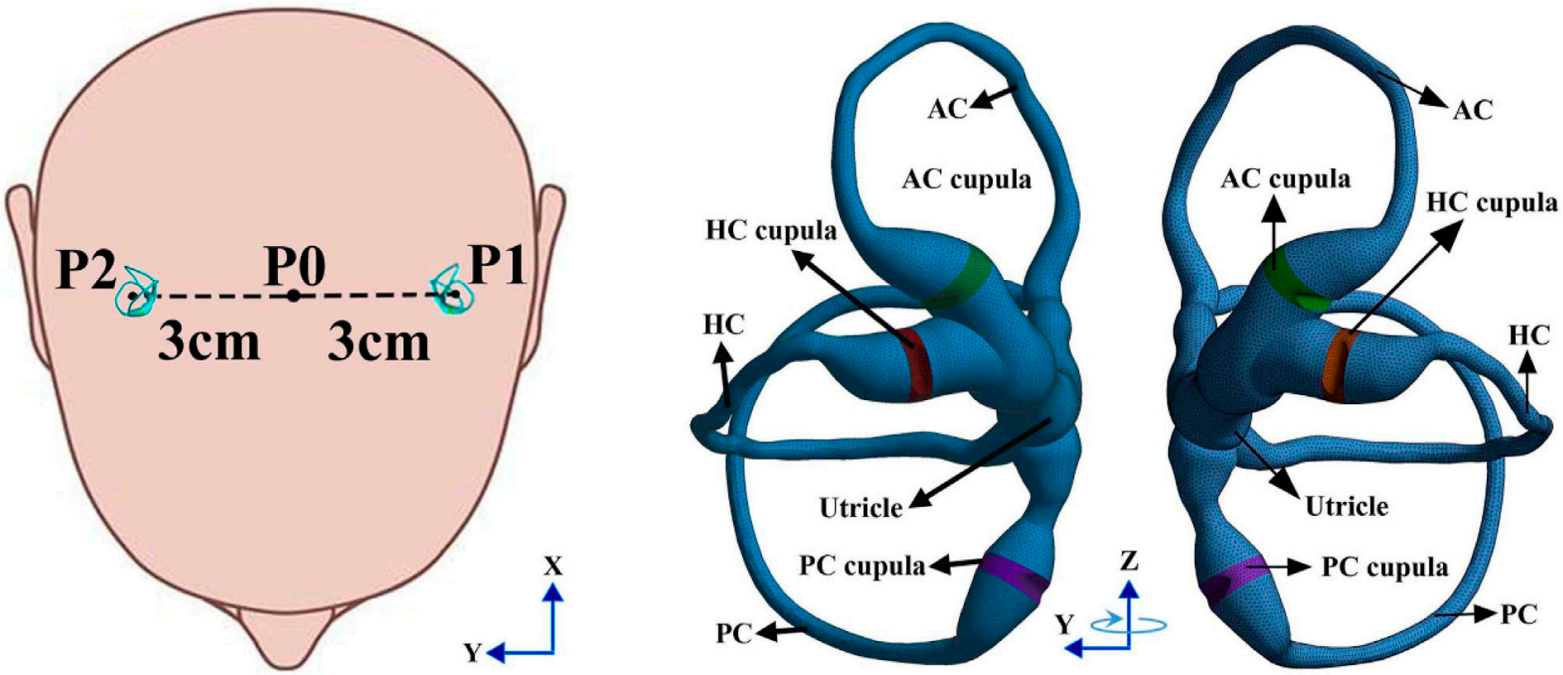
Finite element model of the bilateral SCCs in humans: The rotation radius of the bilateral SCCs was 3 cm, and the centre of rotation was P0.

We built a geometrical model of the bilateral SCCs in the inner ear based on the geometrical parameters provided in the literature (Ifediba et al., 2007). However, the method for defining the geometry of the cupula has not been previously described in Ifediba et al. (2007). Thus, we constructed simplified geometries of the cupulae by trimming the ampullae regions in the SCCs. The cupula was simplified to a cylindrical structure with a thickness of approximately 4.03 × 10^−4^ m (Kassemi et al., 2005; Selva et al., 2009; Goyens et al., 2019), and the connection of the endolymph in the ampullae was blocked. The heights of the cupulae in the anterior SCC, horizontal SCC and posterior SCC were approximately 1.35 × 10^−3^ m, 1.32 × 10^−3^ m, and 1.29 × 10^−3^ m, respectively (i.e., the diameters of the ampullae in SCCs). The geometries of the cristae in the ampullae were constructed by trimming cupular solids in Hypermesh 12.0 based on the geometric parameters provided by Selva et al. (2009). The height of the cristae was approximately 2 × 10^−4^ m which was smaller than that of Selva et al. due to the height of the cupulae in our model being smaller. The fluid region of the endolymph in the unilateral SCCs was meshed with 183 k tetrahedral elements and 39 k nodes. In contrast, the solid region of the cupulae in the unilateral SCCs was meshed with 42 k tetrahedral elements and 9 k nodes. The finite element model of the bilateral SCCs in humans is shown in Figure 2. The physical properties of the endolymph and cupula employed in the numerical model are detailed in Table 1.

**TABLE 1:** Physical properties of endolymph and cupula.

Parameters for physical properties	Value
Endolymphatic density (Kg/m^3^)	1000 (Steer et al., 1967)
Endolymphatic viscosity (Pa s)	0.001 (Boselli et al., 2013)
Cupular density (Kg/m^3^)	1000 (Damiano and Rabbitt, 1996)
Young’s modulus of cupula (Pa)	5.4 (Selva et al., 2009)
Poisson ratio of cupula	0.48 (Damiano, 1993)

In this study, a computational model of bilateral SCCs was constructed using ANSYS Workbench (version 16.0). The specific modeling techniques and parameter configurations were derived from Goyens et al. (2019) (see Supplementary Material). In the Fluent module, we established a fluid dynamics model of the endolymphatic fluid, which is generally considered a Newtonian incompressible fluid (Boselli et al., 2013; Wu et al., 2021b). The rationale for considering the endolymph as an incompressible fluid was detailed in the Supplementary Material. The boundary of the endolymphatic fluid was set to “no slip” walls. In the relative reference frame of the SCCs, the movement of the endolymphatic fluid far from the wall of the SCCs lags behind the movement of the SCCs when they follow the head to undergo angular motion. The behavior of the endolymph can then be defined by the Navier-Stokes equation (Eq. 3·3·I4 on pages 147–148 in Batchelor, 2007; Goyens, 2020):

$$\rho_{f}\frac{\partial v}{\partial t}+\rho_{f}\left(v∙\nabla \right)v=-\nabla P+\mu \nabla^{2}v-2\rho_{f}\Omega \times \left(\Omega \times r\right)-\rho_{f}\frac{\partial \Omega }{\partial t}\times r$$ (1)

where $\rho_{f}$ is the fluid density, v is the fluid velocity vector relative to the velocity of the moving reference frame, *P* is the pressure, *µ* is the dynamic viscosity, **Ω** = (0, 0, *ω*) is the angular velocity vector of the moving reference frame, and r is the radial coordinates of the fluid element.

In addition, a computational model of the cupulae was established in the transient structural module. In previous studies, the cupula was assumed as either simple elastic structures (Buskrik et al., 1976; Oman et al., 1987) or visco-elastic structures (Rabbitt et al., 1994). However, the mechanical properties of the cupula in the human vestibular system have not been experimentally determined thus far. Based on the research by Selva et al. (2009), we set the elastic modulus of the cupula to 5.4 Pa when constructing the solid structural model in ANSYS Workbench. Besides, other studies also assumed the cupula to be a linear elastic structure (Kassemi et al., 2005; Shen et al., 2016). In our investigation, the maximal cupula deformation induced by the simulated rotational stimulus did not exceed 6 μm, which was very small relative to its dimensions (the thickness of the cupula >400 μm). We considered that the cupula deformation was still in the stage of linear elastic deformation. Considering the factors mentioned above, we assumed the cupula to be a linear elastic isotropic structure. When the SCCs are stimulated by rotational movement, the behaviour of the cupula can be defined by the Navier equation:

$$\rho_{s}\frac{\partial^{2}d}{\partial t^{2}}=\nabla ∙\sigma_{s}+{2\rho }_{s}\Omega \times \left(\Omega \times r\right)+\rho_{s}\frac{\partial \Omega }{\partial t}\times r$$ (2)

where $\rho_{s}$ is the cupular density, ***d*** denotes the displacement vector and $\sigma_{s}$ denotes the stress tensor:

$$\sigma_{s}=2\mu \varepsilon +\lambda tr\left(\varepsilon \right)I$$ (3)

where ε **is** the strain tensor, tr represents the trace of a matrix, and μ and λ are the Lamé coefficients related to Young’s modulus *E* and Poisson’s ratio *ν*, described by the following equations:

$$\mu =\frac{E}{2\left(1+\nu \right)}$$ (4)

$$\lambda =\frac{\nu E}{\left(1+\nu \right)\left(1-2\nu \right)}$$ (5)

Eqs 3–5 are cited in Selva et al. (2010).

**FIGURE 3:**
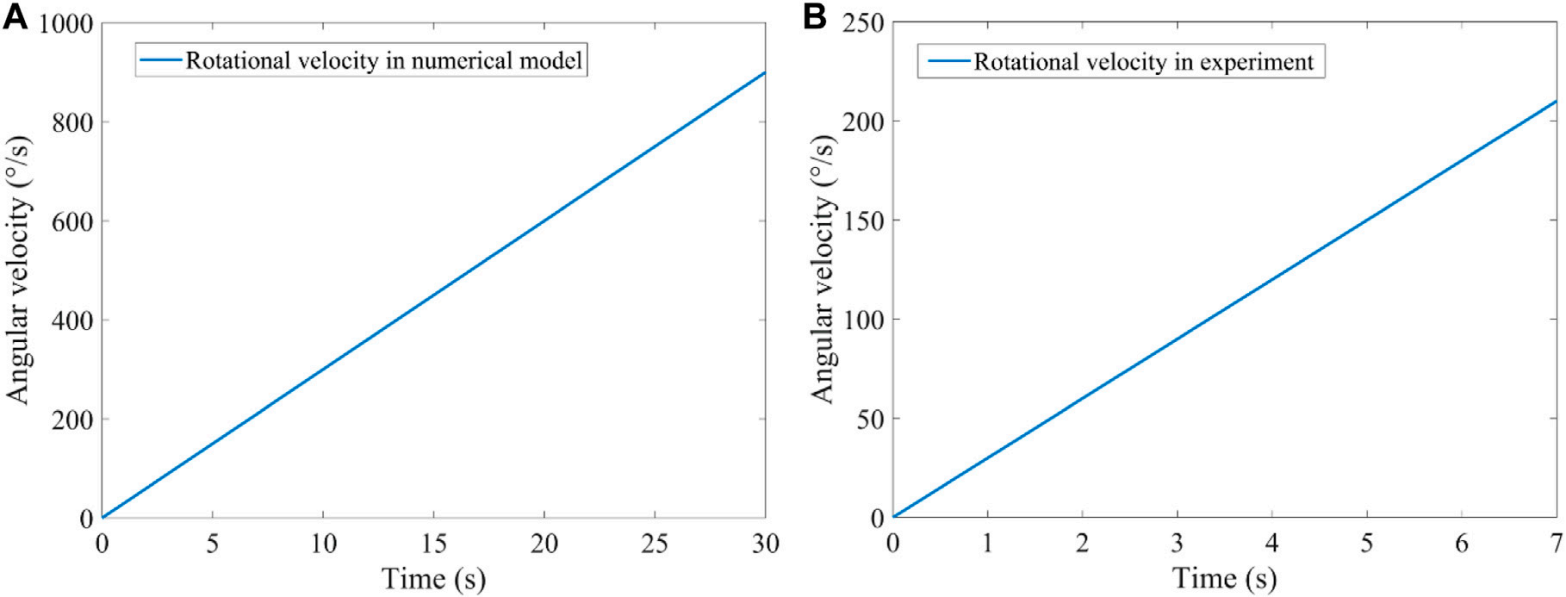
Rotational stimulus. **(A)** Rotational velocity loaded in the numerical model. **(B)** Rotational velocity loaded in the VOR experiment.

As shown in Figure 1B, the rotation axis of the bilateral SCCs passes through point P0 along the positive direction of the *z*-axis when the head is located in different forward-leaning angles of the head. The rotational centre of the bilateral SCCs was P0, and the rotational radii of P0-P1 and P0-P2 were 3 cm. A clockwise angular velocity was applied to the numerical model of the bilateral SCCs. The temporal variation in the magnitude of angular velocity is depicted in Figure 3A. In the system coupling module, the fluid-structure interaction in the SCCs was set. With a maximal number of iterations per time step of 100, the convergence tolerance was set to 0.01. The time step was set as 0.001.

### 2.2 Rotating chair experiments

#### 2.2.1 Volunteers and equipment

**FIGURE 4:**
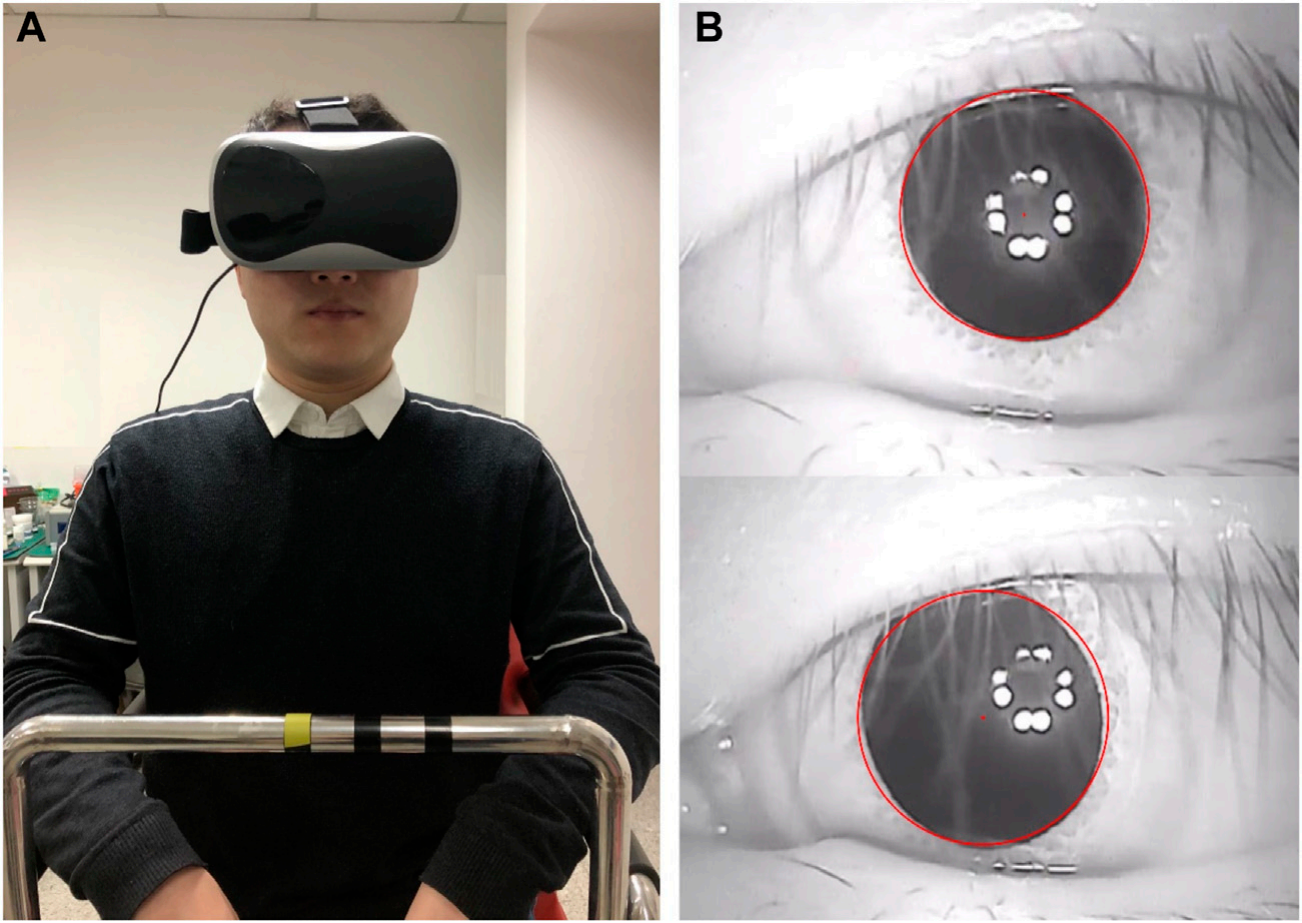
Volunteer participating in the VOR experiment. **(A)** A volunteer sitting on a rotatable chair. **(B)** Tracking and locating the pupil.

Three people volunteered to participate in the rotating chair experiment and provided written informed consent. They were informed of the experimental procedures and allowed to stop the experiments at any time. All volunteers had normal vestibular function. The experimental research was approved by the Biological and Medical Ethics Committee of Dalian University of Technology (registration number 2020–077). All the experimental procedures were performed in accordance with the principles of the Declaration of Helsinki. Each volunteer was asked to wear an eyepatch and sit on a rotatable chair (Figure 4A). A small infrared camera was fixed to the left side of the eye patch to record the movements of the left eye. The camera recorded the videos at a frame rate of 50 fps. A gyroscope was fixed to the right side of the eyepatch to measure and record the instantaneous velocity of the head. The sampling frequency of the gyroscope was 50 Hz.

The eyepatch was tightly fixed to the volunteer’s head to ensure that there was no relative movement between the eyepatch and head. The volunteers wore a seatbelt during the experiment to ensure their safety. They were also required to hold the chair’s armrests with their hands and use the headrest and backrest to increase the physical restraint of the head so as to reduce the relative movement of the head during rotation. In addition, auxiliary signs were marked on the armrest of the chair to keep the volunteers seated in the correct position. A gyroscope was used to adjust the head positions of the volunteers.

#### 2.2.2 Experimental procedure

The volunteers sat on a rotatable chair and were asked to fasten their seatbelt. The volunteers wore an eyepatch which was adjusted so that a small infrared camera could record the movements of the left eye. Each volunteer participated in the rotating chair experiment with their head tilted forward at 0°, 10°, 20°, 30°, 40°, 50°, and 60°. The horizontal angular velocity of the chair was clockwise. The temporal variation in the magnitude of angular velocity is illustrated in Figure 3B. All experiments were performed in a dark room to eliminate any interference from eye movement. When the same volunteer participated in multiple experiments, they were required to rest for at least 30 min before commencing the next session.

#### 2.2.3 Nystagmus data processing

The videos of the eye movements recorded during the experiments were processed using MATLAB R2017b software. The pupil centre was then tracked and located using an image processing method (see Figure 4B). To reduce the statistical error, the first and last nystagmus data obtained were removed. We also discarded the nystagmus data from when the volunteers blinked. The SPV of the volunteers was calculated based on a method provided in the study by Wu et al. (2020). Moreover, the average SPV per second was calculated and used as the SPV of the nystagmus per second.

## 3 Results

### 3.1 Biomechanical response in the SCCs with a normal head position

**FIGURE 5:**
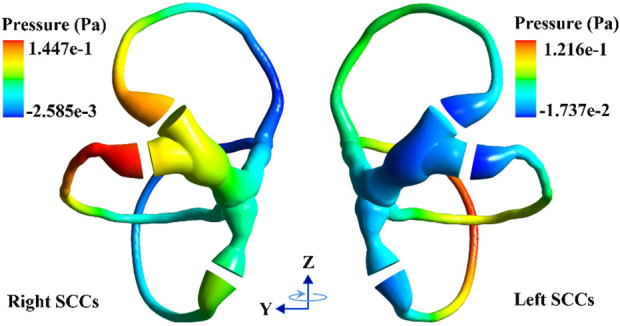
The pressure distribution of endolymph with a normal head position.

**FIGURE 6:**
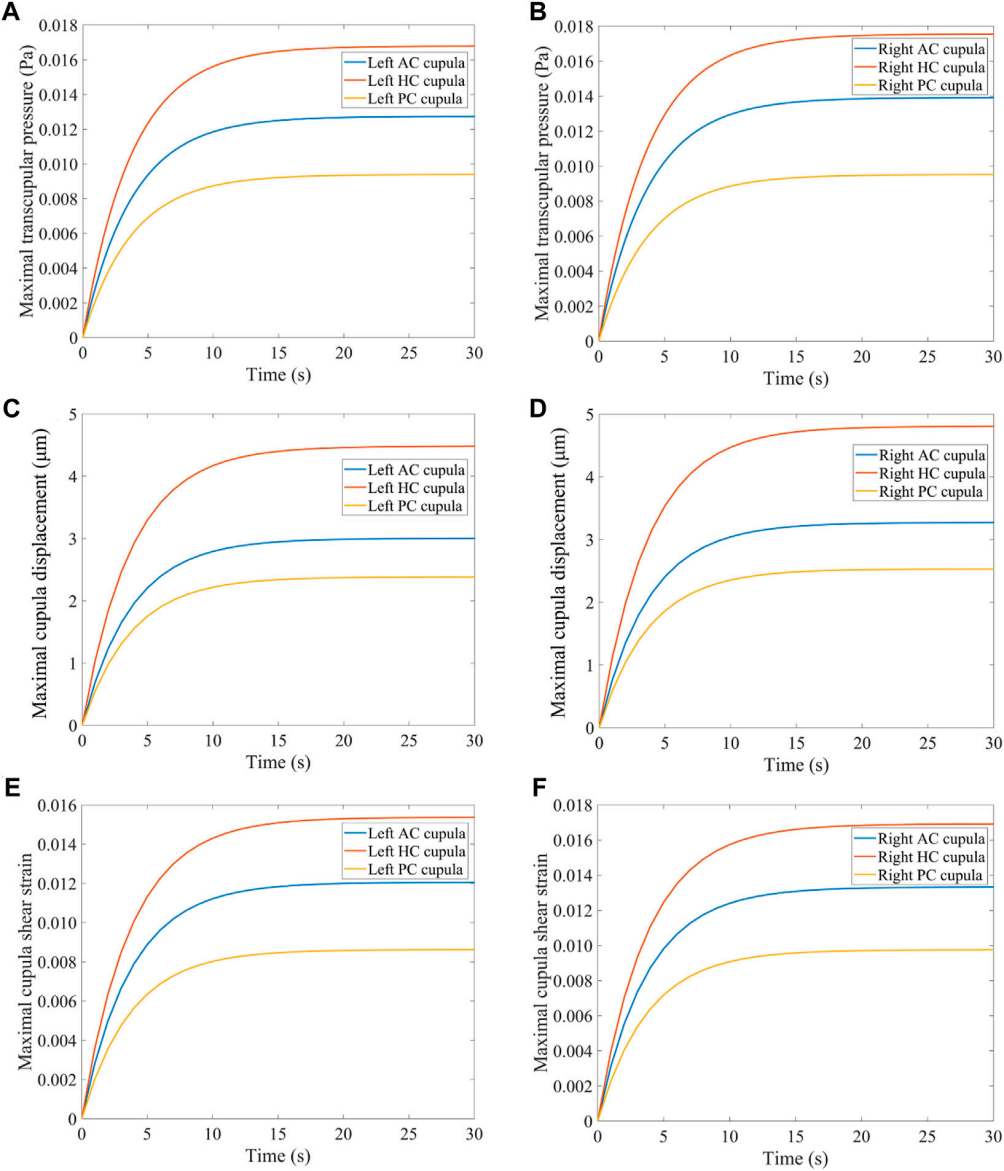
Biomechanical responses in the bilateral SCCs. **(A)** Maximal transcupular pressure changes over time in the left SCCs. **(B)** Maximal transcupular pressure changes over time in the right SCCs. **(C)** Maximal cupula displacement changes over time in the left SCCs. **(D)** Maximal cupula displacement changes over time in the right SCCs. **(E)** Maximal cupula shear strain changes over time in the left SCCs. **(F)** Maximal cupula shear strain changes over time in the right SCCs.

When the head experiences angular acceleration, the distribution of the endolymphatic fluid pressure gradient in the SCCs is generated along the rotational direction because of inertia (see Figure 5). Within 0–30 s of accelerated rotation, the transcupular pressure was found to first increase and then gradually stabilise (Figures 6A, B). Meanwhile, the cupulae were deflected by the transcupular pressure in the bilateral SCCs. As shown in Figures 6A–F, the maximal displacement and shear strain of the cupula in each SCC were found to be consistent with the variation trend of the corresponding transcupular pressure. From 0 to 20 s, the maximal displacement and shear strain of the cupulae in the bilateral SCCs were found to increase. During the 20–30 s period, the elastic force of the cupula in each SCC was balanced by the corresponding transcupular pressure, indicating that the maximum displacement and shear strain of the cupulae had reached a stable state. Maximal cupula displacement was found to occur at the centre of the cupula, whereas the maximal cupula shear strain appeared near the centre of the crista surface. When the head rotated at a constant velocity, the endolymphatic pressure difference caused by the angular acceleration disappeared, which would no longer deflect the cupulae. The cupula gradually returned to its resting position under the combined action of its own elastic restoring force and the viscous resistance of the endolymph. The cupula time constant, measured at 3.7 s in the numerical model of the SCCs (see Supplementary Material), reflected the time course in which cupula displacement increased with the elevation of angular velocity of the head under constant angular acceleration. The cupula time constant, an intrinsic parameter of the endolymph-cupula system in SCCs, is only related to the geometry of the SCCs and the physical properties of the endolymph and cupula. Therefore, the cupula time constant remains the same when the SCCs are rotated under different forward-leaning head positions.

### 3.2 Biomechanical responses of SCCs under different forward-leaning angles

**FIGURE 7:**
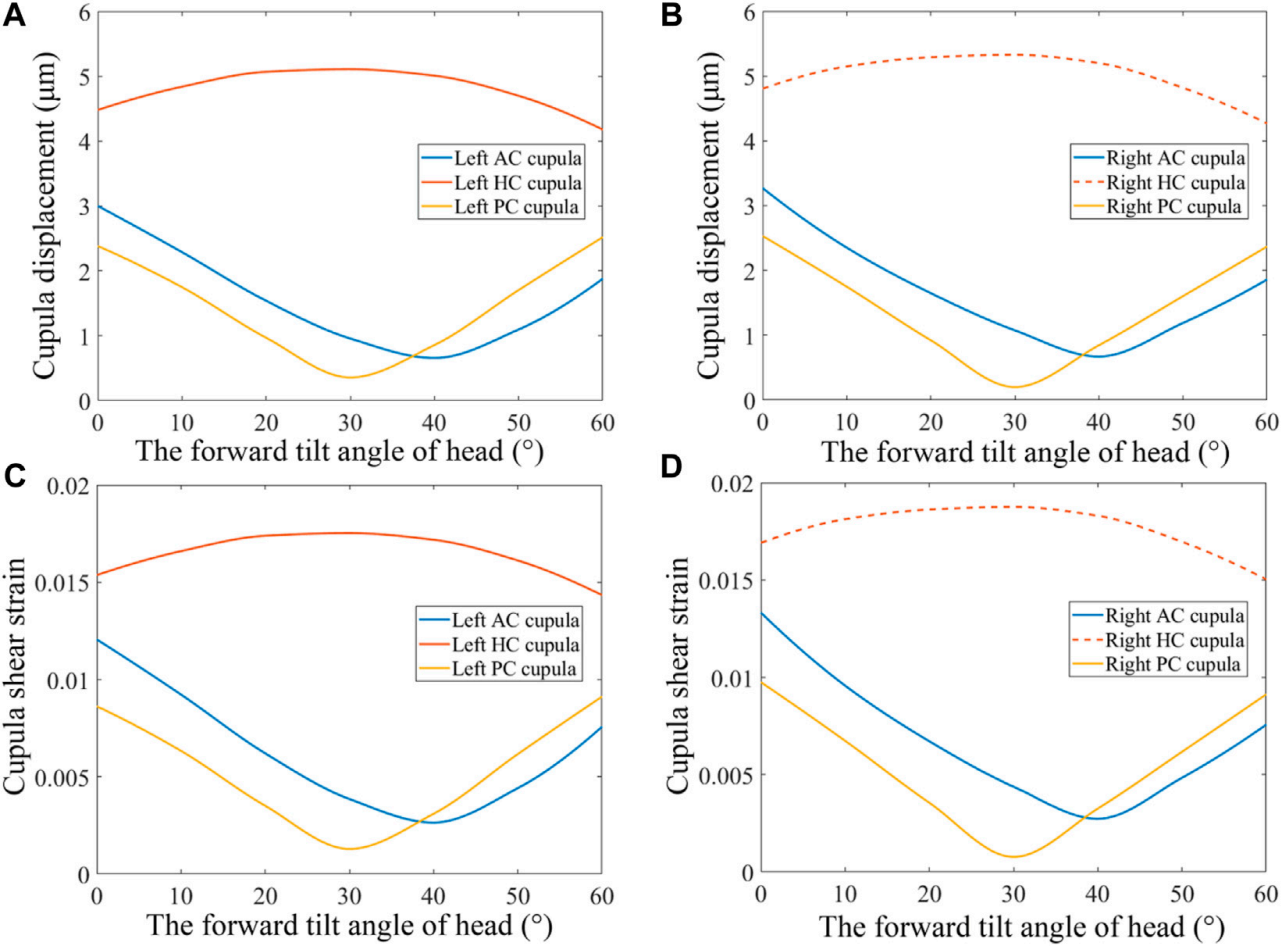
Biomechanical responses in the bilateral SCCs under different forward-leaning angles of the head. **(A)** Maximum cupula displacement in the left SCCs under different forward-leaning head positions. **(B)** Maximum cupula displacement in the right SCCs under different forward-leaning head positions. **(C)** Maximum cupula shear strain in the left SCCs under different forward-leaning head positions. **(D)** Maximum cupula shear strain in the right SCCs under different forward-leaning head positions.

**FIGURE 8:**
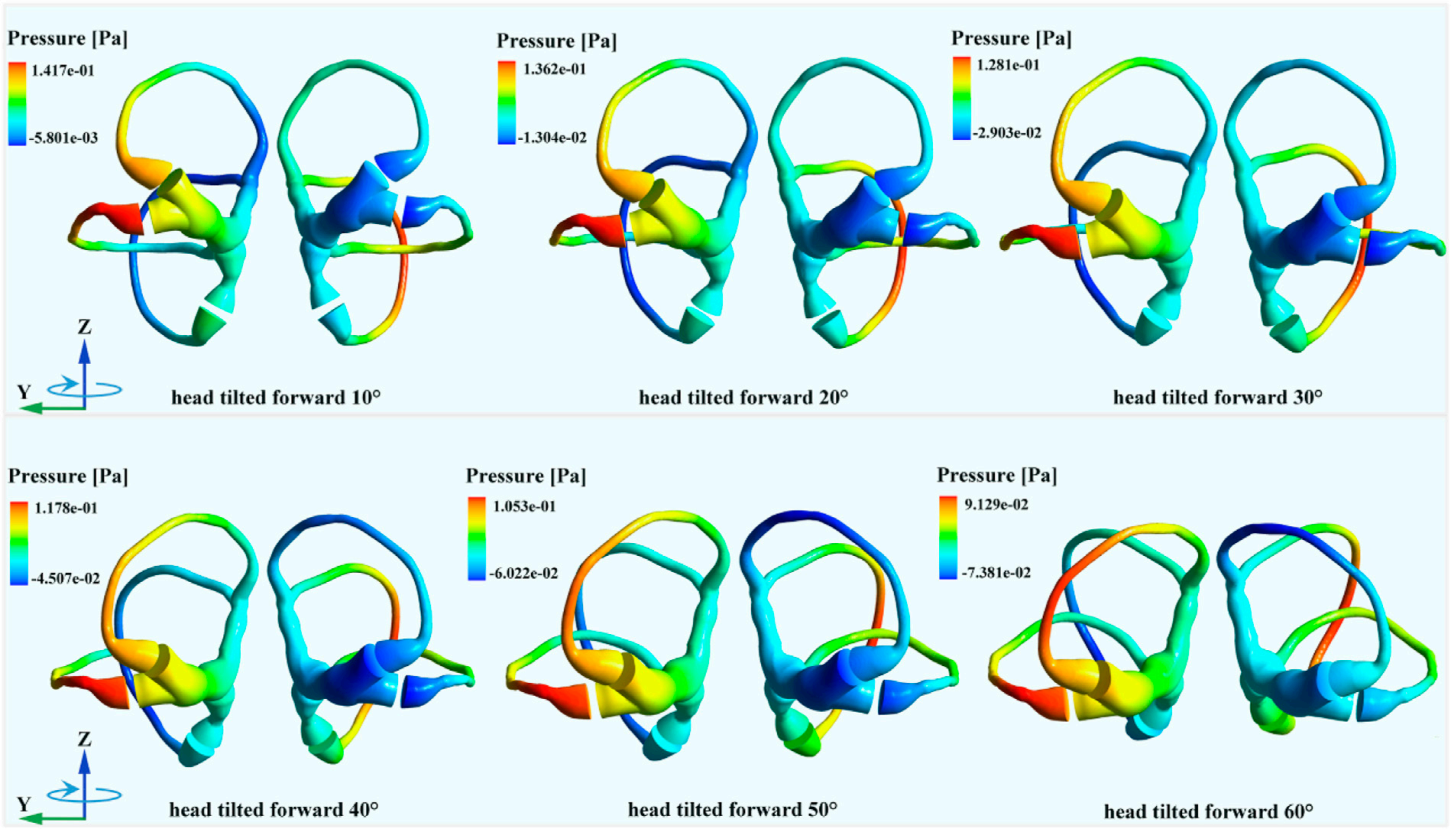
The endolymphatic pressure distribution in the bilateral SCCs under different forward-leaning angles of the head (more detailed legends were shown in Supplementary_Material).

**FIGURE 9:**
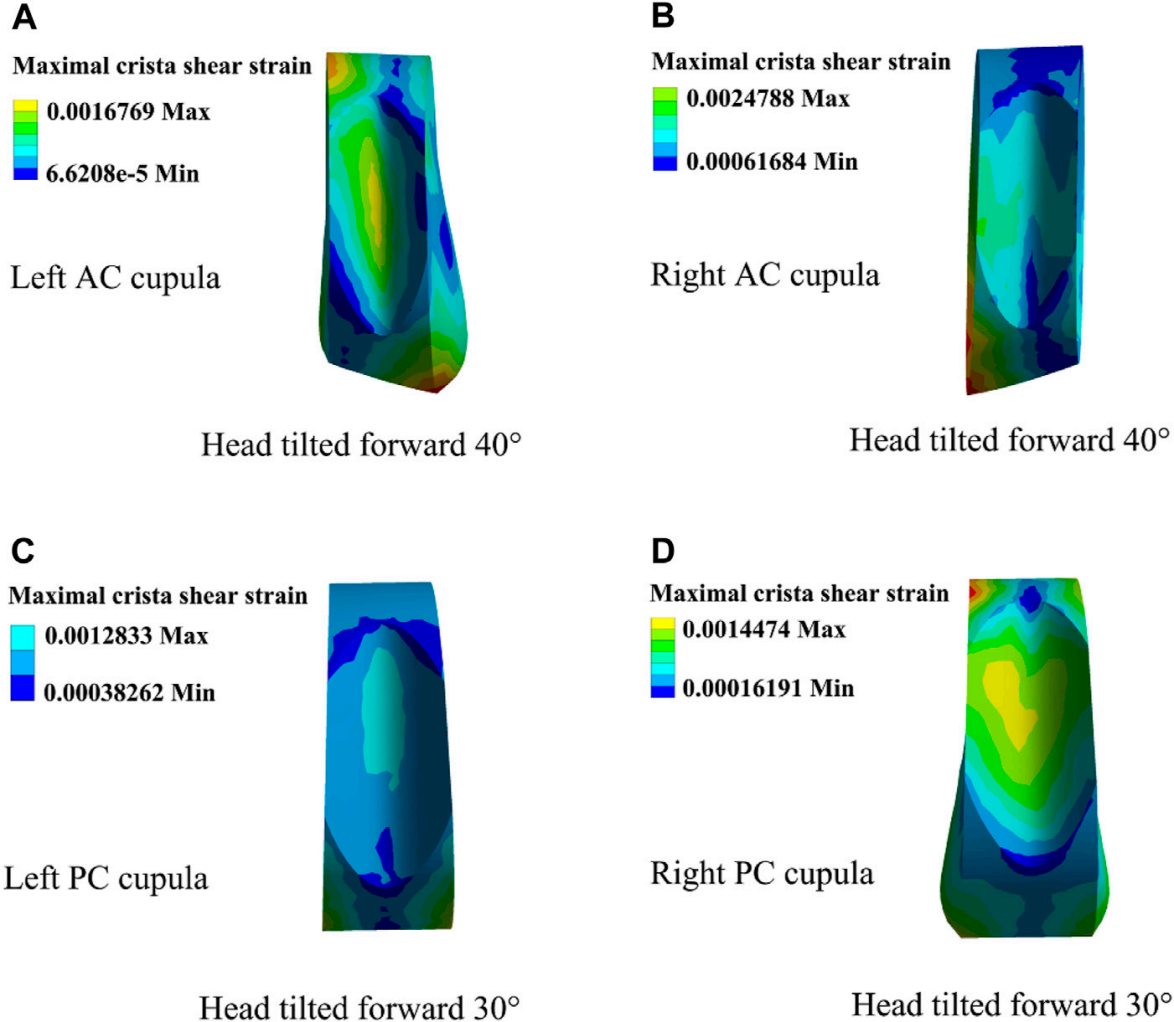
Cupula shear strain in the horizontal and anterior SCCs under different forward-leaning angles of the head. **(A)** Left AC cupula shear strain when the head was tilted forward 40°. **(B)** Right AC cupula shear strain when the head was tilted forward 40°. **(C)** Left PC cupula shear strain when the head was tilted forward 30°. **(D)** Right PC cupula shear strain when the head was tilted forward 30°.

The changes in cupula displacement and shear strain with different forward-leaning angles are shown in Figure 7. With an increase in the forward-leaning angle, the maximal cupula displacement, and shear strain in the horizontal SCCs gradually increased and reached a maximum when the head was titled approximately 30° forward, after which they gradually decreased. However, when the forward-leaning angle of the head increased, the maximal cupula displacement, and shear strain in the anterior SCCs gradually decreased and reached a minimum when the head was tilted approximately 40° forward, after which they gradually increased. For the posterior SCCs, the maximal cupula displacement, and shear strain also decreased gradually with an increase in the forward-leaning angle and reached a minimum when the head was titled forward approximately 30°, after which they gradually increased. Different forward-leaning head positions induced different fluid pressure distribution in the bilateral SCCs (see Figure 8). It is worth mentioning that the endolymphatic fluid pressure on both sides of the cupula in the anterior SCC of the left ear was almost equal leading to the minimum crista shear strain when the head was tilted forward by approximately 40° (see Figure 9A). Due to the negative endolymphatic fluid pressure on both sides of the cupula in the anterior SCC of the left ear, the cupula expanded. In contrast, concerning the anterior SCC of the right ear, the endolymphatic fluid pressures on both sides of the cupula were nearly equal, resulting in the minimum crista shear strain (Figure 9B). The cupula in the anterior SCC of the right ear compressed because of the positive endolymphatic fluid pressure on both sides. Regarding the posterior SCC of the left ear, the endolymphatic fluid pressures on both sides of the cupula were almost equal inducing the minimum crista shear strain (Figure 9C). The cupula in the posterior SCCs of the left ear compressed because the endolymphatic fluid pressure on both sides of the cupula was positive. When the head was tilted forward 40°, the endolymphatic fluid pressure on both sides of the cupula in the posterior SCC of the right ear was almost equal causing the minimum crista shear strain (Figure 9D). Since the endolymphatic fluid pressure on both sides of the cupula in the posterior SCC of the right ear was negative, the cupula in the posterior SCC of the right ear expanded.

### 3.3 Nystagmus characteristic with a normal head position

**FIGURE 10:**
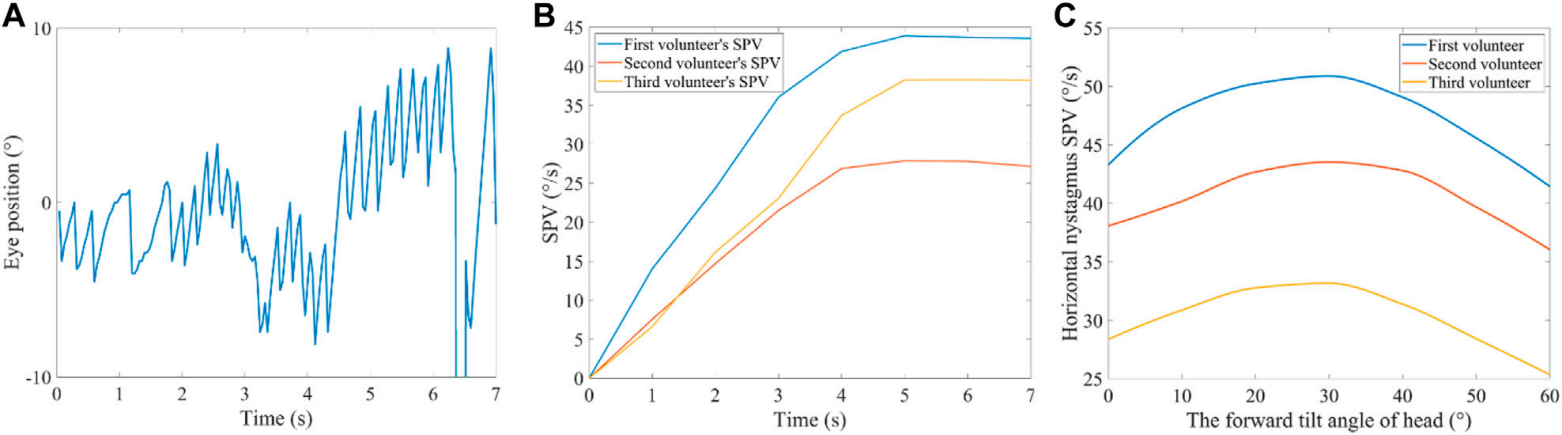
Nystagmus in the VOR experiment. **(A)** Horizontal nystagmus of a volunteer with a normal head position. **(B)** Nystagmus SPV in the time domain of the three volunteers with a normal head position. **(C)** Nystagmus SPV of the three volunteers with their heads tilted 0°, 10°, 20°, 30°, 40°, 50°, and 60°, forward.

When the volunteer’s heads were rotated with a horizontal angular acceleration of 30°/s^2^, the horizontal nystagmus was measured with a normal head position. No evidence of vertical or torsional nystagmus was found. Figure 10A shows the horizontal nystagmus trajectory of a volunteer. The trajectory curves with positive slopes were in the slow-phase of nystagmus, while those with negative slopes were in the fast phase of nystagmus. An interrupted part of the trajectory curve indicates that the volunteers blinked. In the fast and slow phases of nystagmus, the absolute value of the slope of the trajectory curve represents the velocity of the eye movements. The SPV of the three volunteers gradually increased from 0 s to 5 s (Figure 10B). When the time was between 5 and 7 s, the SPV of the three volunteers stabilised, indicating that the neural signal transmitted to the brain responsible for triggering the nystagmus SPV had reached saturation. During the constant rotational acceleration of 5–7 s, the average SPV of the three volunteers was 43.7°/s, 38.3°/s, and 27.5°/s, respectively. The nystagmus SPV differed among different volunteers under the stimulation of the same rotational acceleration, which might be caused by individual differences. In addition, no obvious vertical nystagmus or torsional nystagmus was observed in the three volunteers.

### 3.4 Nystagmus SPV under different forward-leaning angles

The experimental results showed that the three volunteers had horizontal nystagmus under different forward-leaning angles of the head (including the head tilted 0°, 10°, 20°, 30°, 40°, 50°, and 60° forward) but no obvious vertical nystagmus or torsional nystagmus. Considering that the volunteers’ nystagmus SPV reached a steady state within 5–7 s, we calculated the average of the volunteers’ nystagmus SPV during 5–7 s as stable nystagmus SPV. Figure 10C shows the average nystagmus SPV of the three volunteers under different forward-leaning angles of the head, including 0°, 10°, 20°, 30°, 40°, 50°, and 60°. We found that the horizontal nystagmus SPV of the three volunteers gradually increased with an increase in the forward-leaning angles of the head, reached a maximum when the head was tilted forward approximately 30°, and then gradually decreased.

## 4 Discussion

When the head experienced angular acceleration, the changes in the forward-leaning angles of the head resulted in different distributions of the endolymphatic fluid pressure gradients in the SCCs, affecting the transcupular pressure and generating different cupula displacement and shear strain. The corresponding vestibular SCCs produced excitation, and the combined action of the excited SCCs induced horizontal nystagmus under different forward-leaning head positions (including the head tilted at 0°, 10°, 20°, 30°, 40°, 50°, and 60°), but no obvious vertical nystagmus or torsional nystagmus.

When the head forward angle was between 0° and 30°, the cupula in the horizontal SCC of the right ear was deflected to the utricle side, and the cupulae in the anterior SCC of the left ear and posterior SCC of the left ear were deflected to the canal side. According to Ewald’s law (Ewald, 1892), the horizontal SCC of the right ear, the anterior SCC of the left ear, and the posterior SCC of the left ear were excited. In contrast, the horizontal SCC of the left ear, the anterior SCC of the right ear, and the posterior SCC of the right ear were inhibited. Based on Eggers et al. (2019), the excitation of each SCC influences the extraocular muscles and triggers corresponding eye movements (see Supplementary Material for more detail). Excitation of the horizontal SCC in the right ear would cause the eyeball to move horizontally to the left in the volunteers. Excitation of the anterior SCC in the left ear causes the eyeball to turn up and rotate clockwise, while excitation of the posterior SCC in the left ear causes the eyeball to turn down and rotate clockwise. When the head was tilted forward 0°, the maximum cupula displacement in the horizontal SCC of the right ear was the largest. The horizontal SCC of the right ear was more excited than the other SCCs, resulting in obvious horizontal nystagmus in the volunteers. Compared with the maximal cupula displacement in the horizontal SCC of the right ear, the maximal cupula displacement in the anterior and posterior SCCs of the left ear was smaller. Moreover, the combined excitation of the anterior and posterior SCCs in the left ear would weaken the upward and downward movements of the eyeball. Thus, no obvious vertical nystagmus was observed in the volunteers. Although the direction of the rotational eye movement caused by excitation of the anterior and posterior SCCs in the left ear was the same, the maximal cupula displacement in the anterior and posterior SCCs of the left ear was so small that the combined excitation of the anterior and posterior SCCs of the left ear was not sufficient to produce obvious torsional nystagmus in the volunteers. With an increase in the forward-leaning angles of the head, the maximal cupula displacement in the horizontal SCCs gradually increased, while the maximal cupula displacement in the anterior and posterior SCCs of the left ear gradually decreased. When the head was tilted forward 30°, the transcupular pressure in the posterior SCCs was approximately equal, which resulted in no deflection of the cupula. The posterior SCCs were then in a resting state. However, the cupula in the left PC was compressed due to positive fluid pressure on both sides, while the cupula in the right PC was expanded because of the negative fluid pressure on both sides. For the horizontal SCCs, the cupula displacement reached a maximum that induced maximal SPV in the volunteers.

When the forward-leaning angle of the head was between 30° and 60°, the state of excitation and inhibition in the posterior SCCs changed. The cupula in the posterior SCC of the left ear was deflected to the side of the utricle by the action of transcupular pressure, which induced inhibition. Regarding the posterior SCC of the right ear, the cupula was deflected to the side of the canal by transcupular pressure, which caused excitation. As for the anterior SCCs, they were in a resting state when the head was tilted forward 40°. In addition, the state of excitation and inhibition in the anterior SCCs changed when the forward-leaning angle of the head was between 40° and 60°. The cupula in the anterior SCC of the left ear was deflected to the side of the utricle by transcupular pressure, which induced inhibition. In the anterior SCC of the right ear, the cupula was deflected to the side of the canal by transcupular pressure, which caused excitation. According to a previous report (Eggers et al., 2019), excitation of the anterior SCC in the right ear caused the eyeball to turn up and rotate anticlockwise. Excitation of the posterior SCC in the right ear induced the eyeball to turn down and rotate anticlockwise. When the head was tilted forward between 30° and 60°, the maximal cupula displacement in the horizontal SCC of the right ear decreased as the forward-leaning angle of the head increased, which resulted in a decrease in the horizontal nystagmus SPV in the three volunteers. Regarding the posterior SCC in the right ear, the maximal cupula displacement gradually increased. In contrast, in the anterior SCC in the right ear, the maximal cupula displacement gradually decreased, reached a minimum when the head was tilted 40° forward, and then gradually increased. However, the maximal cupula displacement in the excited anterior SCC of the right ear was still small and partially offset by the maximal cupula displacement in the excited posterior SCC of the right ear. Consequently, the excitation causing the eyeball to move up and down became so weak that there was no significant vertical nystagmus in the volunteers. In addition, there was no obvious torsion nystagmus in the volunteers because the maximal cupula displacement in the excited anterior and posterior SCCs was also very small, which led to the combined effects of the excitation of the anterior and posterior SCCs in the right ear being insufficient to induce torsion nystagmus.

When employing the rotating SCCs as a reference frame, we compared viscous, inertial, and convective terms within the fluid domain under normal head position (see Supplementary Material for details). The maximum magnitudes of inertial and convective terms were comparable in the regions of narrow SCCs, while the convective term could be considered negligible. At the initial moment, there was an increase in inertial forces in the regions of narrow SCCs. As time increased, the inertial force decreased to a negligible level, and the viscous force increased, while the convective forces could be considered negligible in the time domain. This phenomenon might arise due to the deformation of the cupula caused by the pressure gradient, leading to relative flow in the regions of narrow SCCs. These results we obtained were similar to those in the study by Goyens et al. (2019).

Typically, each SCC has a synergistic SCC on the contralateral plane to sense the rotation of the corresponding plane (Shen et al., 2020). For example, the left and right horizontal SCCs constitute a pair of SCCs approximately in the same plane; the left anterior and right posterior SCCs constitute a pair of SCCs approximately in the same plane, and the right anterior and left posterior SCCs constitute a pair of SCCs approximately in the same plane. Previous studies have shown that cupula displacement in each pair of SCCs and results of VOR experiments conforms to the law of cosine and exhibits a similar trend of change under different left-leaning head positions (Blanks et al., 1975; 1985; Estes et al., 1975; Dickman, 1996; Rabbitt, 1999; 2019; Raphan and Cohen, 2002; Della Santina et al., 2007). However, the real geometry of the SCCs in the inner ear of humans is not an ideal ring-shaped structure, and the biomechanical response of the cupula is affected by the geometry of the SCCs and the fluid coupling between the canals (Rabbitt, 1999). The results of the current study suggest that when the rotation axis is located in the mid-sagittal plane, making the bilateral SCCs symmetrical to each other, the biomechanical responses of the cupula in each pair of symmetrical SCCs exhibit approximately the same trends. This is attributed to the horizontal angular acceleration, when decomposed into the direction of each SCC, exerting the same magnitude and direction on each pair of symmetrical SCCs. Consequently, the distribution of endolymphatic pressure gradients along the acting SCCs is very similar. When the bilateral SCCs were asymmetrical with respect to the mid-sagittal plane, the magnitudes of the angular acceleration components were equal. However, the direction differed after decomposing the horizontal angular acceleration on the SCCs in the same plane into the directions of the other SCCs. Therefore, the distribution of the endolymphatic pressure gradient along the acting SCCs was different, which resulted in a significant difference in the biomechanical responses of the cupula in the SCCs located in approximately the same plane. The relative spatial positioning of the geometrical structures of bilateral SCCs, with respect to the mid-sagittal plane where the rotation axis was located, influenced the pressure distribution of the endolymph within the SCCs, subsequently determining the cupula/shear strain. This quantitative investigation into the unique spatial positioning of the SCCs provided in-depth insights into the biomechanical mechanisms of the SCCs and their effects on function. It held important reference value for clinical research aimed at alleviating vestibular diseases caused by spatial orientation.

In this study, the horizontal nystagmus SPV was induced by the cupula displacement/shear strain in the horizontal SCC of the right ear. When the forward-leaning angle of the head increased from 0° to 30°, the maximum cupula displacement in the horizontal SCC of the right ear exhibited an increment of 5 μm, rising from approximately 4.8 μm to about 5.3 μm. The nystagmus SPV showed a significant increase for all three volunteers: the first volunteer’s SPV increased by about 8°/s, the second volunteer’s SPV increased by approximately 5°/s, and the third volunteer’s SPV increased by about 5°/s. The pronounced changes in SPV of nystagmus resulting from subtle cupula displacements were attributed to the presence of numerous sensitive afferent nerves in the crista region (Eatock and Songer, 2011). Consequently, even small displacements of the cupula can trigger significant eye movements. These results have a certain reference value for clinical applications. However, the geometrical morphology of SCCs varies among individuals (Cox and Jeffery, 2010), which quantitatively influences the cupula in response to angular velocity experienced by the head. The current numerical model in this study might not accurately capture the intricate and individualized nature of the vestibular system among volunteers because the construction of geometrical model was based on anatomical parameters from another individual. The vestibular system will exhibit inter-individual variability in terms of anatomical structure and response magnitude. In fact, noticeable differences in nystagmus SPV among the three volunteers existed under the same angular velocity stimulus due to individual variability. Furthermore, there were variations in the increased nystagmus SPV among the volunteers as the forward-leaning angle increased from 0° to 30°. However, the patterns of biomechanical mechanism in SCCs detecting angular motion exhibited consistent among individuals with normal vestibular function. The geometrical model of SCCs in this study was constructed by the anatomical parameters of a healthy individual. Therefore, the regularities manifested in the numerical results of this study were relevant to these volunteers. However, the quantitative magnitudes of the outcomes may not precisely correspond to responses of their actual SCCs. In summary, the numerical results presented in this study had significant reference value for clinical applications. but were not directly applicable to clinical practice.

## 5 Conclusion

We quantitatively investigated the responses of human SCCs using the numerical simulation of fluid-structure interaction and VOR experiments under different forward-leaning angles of the head, including 0°, 10°, 20°, 30°, 40°, 50°, and 60°. The horizontal nystagmus SPV and corresponding biomechanical responses of the cupula increased with the forward-leaning angles of the head, reached a maximum when the head was tilted 30° forward, and then gradually decreased. Besides, there was no obvious vertical or torsional nystagmus in the VOR experiments. In the numerical model of bilateral SCCs, the biomechanical responses of the cupula in a pair of anterior SCCs showed the same trends; they decreased with the forward-leaning angles, reached a minimum when the head was tilted 40° forward, and then gradually increased. The biomechanical responses of the cupula in a pair of posterior SCCs also showed the same trend; they decreased with the forward-leaning angles, reached a minimum when the head was tilted 30° forward, and then increased gradually. The reason for these numerical results was that the bilateral SCCs were mutually symmetrical with respect to the mid-sagittal plane containing the axis of rotation. This symmetry resulted in the biomechanical responses of the cupula in each pair of symmetrical SCCs exhibiting the same tendencies under different forward-leaning angles of the head. This quantitative investigation into the unique spatial positioning of the SCCs provided in-depth insights into the biomechanical mechanisms of the SCCs and their effects on function. It provided a reliable numerical basis and played an important role in clinical research for alleviating vestibular diseases caused by spatial orientation.

## Data Availability

The original contributions presented in the study are included in the article/Supplementary Material, further inquiries can be directed to the corresponding authors.
