# Supplementary material for: Investigation on biomechanical responses in bilateral semicircular canals and nystagmus in vestibulo-ocular reflex experiments under different forward-leaning angles
Source: Front Bioeng Biotechnol. 2024 Feb 7;12:1322008. doi: 10.3389/fbioe.2024.1322008 (PMC10879882; doi:10.3389/fbioe.2024.1322008)
Supplement: Supplementary file 1 [file DataSheet1.DOCX]

Supplementary Material

# Supplementary Data

**(1) The method of modeling geometries of cupulae and cristae**

**(2) The method of constructing computational model for endolymph flow and cupulae**

**(3) The method of measuring slow-phase eye movements**

**(4) Additional VOR experiment to determine the time of the volunteer’s SPV becoming stable**

**(5) The consequences of exciting/inhibiting signals on the VOR**

**(6) The effects of different rotation radius in the model and VOR experiments**

**(7) Cupula time constant**

**(8) Endolymphatic pressure distribution under different forward-leaning head positions**

**(9) Comparison on the viscous, inertial, and convective terms in the regions of narrow SCCs**

**(10) References**

1. **The method of modeling geometries of cupulae and cristae**

The geometry of the SCCs was constructed based on the geometric parameters provided by Ifediba et al. (2007). However, the method of defining the geometry of the cupula was not described in this literature. Thus, we constructed simplified geometries of the cupulae by trimming solid of ampullae regions in SCCs. The cupula is simplified to a cylindrical structure which had the thickness of approximate 4.03×10^-4^m (Kassemi et al., 2005; Selva et al., 2009; Goyens et al., 2019) and blocked the connection of endolymph in ampullae. The height of the cupulae in anterior SCC, horizontal SCC, and posterior SCC was approximately 1.35×10^-3^m, 1.32×10^-3^m, and 1.29×10^-3^m, respectively (i.e., the diameters of the ampullae in SCCs). Considering the sensory hair cell bundles locating at the top of the crista, the cupula shear strain at the crista surface could represent the deflection of the sensory hair cell bundles. Thus, we constructed the geometries of the cristae in ampullae by trimming cupular solids in HyperMesh (version 12.0) based on geometric parameters provided by Selva et al. (2009). The height of cristae was approximately 2×10^-4^m which was reduced appropriately because the height of cupulae in our model was smaller than that of (Selva et al., 2009). The model of three cupulae are shown in Figure S1.

1. **The method of constructing computational model for endolymph flow and cupulae**

We constructed the model in ANSYS Workbench, and the specific modeling techniques and parameter configurations were derived from Goyens et al. (2019). Firstly, we imported the fluid domain grids and solid domain grids into module of Fluid Flow (Fluent) and Transient Structural respectively. Secondly, we used Fluent for modeling endolymph flow and Transient Structural for constructing the finite element model of cupulae. Lastly, the module of System Coupling was used for constructing the fluid-structure interaction. The fluid region of the endolymph in the unilateral SCCs was meshed with 183 k tetrahedral elements and 39 k nodes. The solid region of the cupulae in the unilateral SCCs was meshed with 42 k tetrahedral elements and 9 k nodes.

In module of Fluid Flow (Fluent), a laminar viscous model was selected due to the low Reynolds number (with a maximum local Reynolds number of approximately 0.08 in this model). We employed a pressure-based solver with second-order spatial discretization and first-order implicit time integration for the temporal discretization. The pressure-velocity coupling scheme adopted was implemented using a "simple" approach. The endolymph was set to incompressible liquid; its density and viscosity are 1000 kg/m3, and 0.001 Pa s, respectively. The compressibility or incompressibility of endolymph within the SCCs is fundamentally determined by the physical properties of the endolymph itself. Under typical physiological conditions, the endolymph in the SCCs is commonly regarded as an incompressible fluid. This incompressibility is attributed to the exceedingly small molecular distance within the liquid, presenting a notable challenge for further reduction in molecular distance and consequential volume reduction. However, the assumption of endolymph incompressibility has inherent limitations. For example, in scenarios involving rapid liquid flow or exposure to exceedingly high pressures, changes in liquid pressure and density cannot be disregarded, and the compressibility of the liquid may become significant. Nevertheless, such extreme conditions are infrequent in the normal physiological functioning of the vestibular system. Then, under normal physiological conditions, it was reasonable to consider the endolymph as an incompressible fluid. Besides, the boundary conditions of the walls of endolymph were set to ‘No Slip’ (i.e., the membranous walls are equivalent to the walls of endolymph). The Frame Motion was used, and Zone Motion Function was set by the “Define Zone Motion” macro of user-defined function to accomplish head rotation, which allow for the walls of endolymph moving together with rotating reference frame. In the absolute reference frame, the walls of endolymph are rotating. In the “relative reference frame”, the walls of endolymph remain stationary because the reference frame moves together with the walls, and then the endolymph flow is considered as movement relative to the reference frame as well as the walls. Moreover, we used the function of smoothing and remeshing in the Dynamic Mesh because cupulae deformation was transferred to neighbouring endolymph elements. As Goyens et al described, a coefficient-based scale factor of 1.5 in solution stabilization was necessary to be set to make the model stable. The time step size was set to 0.001 with 100 of maximum iterations per time step, and the residuals for continuity and x-, y-, and z-velocity were 1e-5,1e-7,1e-7 and 1e-8, respectively.

In module of Transient Structural, material properties of the cupulae were set to the density of 1000kg/m^3^ (Rajguru et al., 2004; Kassemi et al., 2005), the Young’s modulus of 5.4 Pa (Selva et al., 2009), and the Poisson ratio of 0.48 (Kassemi et al., 2005). A boundary condition of “rotational velocity” was used for describing cupulae movement induced by head rotation. Besides, all surfaces of cupulae except those interact with endolymph were set to fixed support boundary condition.

In module of System Coupling, fluid–structure interaction of endolymph and cupula was constructed. As Goyens et al. (2019) described, the force calculated for every endolymph element by Fluent was transferred to the neighbouring cupulae element. Then, the cupulae displacement induced by the corresponding force in Transient Structural was transferred to the neighbouring endolymph elements which moved in accordance with cupulae elements. The cycle was repeated until calculation reached the default convergence value of 0.01. The time step was set to 0.001 with 100 of maximum iterations per time step.

Besides, we refined the mesh in the fluid domain of endolymph, using 492 k tetrahedral elements and 103 k nodes. We calculated the refined model and original model by loading 30°/s^2^ for 30 s. When the number of elements was more than doubled, the results of fluid movement and cupula deformation/shear strain were almost the same. This observation suggested that the initially chosen number of elements for the original model was appropriate to describe the fluid movement.

While our simulations aimed for a high level of detail, it's important to clarify that they might not strictly qualify as DNS due to the selection of laminar viscous model in Fluent and limitations in computational resources. Nevertheless, the chosen resolution allowed for a comprehensive representation of the dynamic interplay between the fluid and the structure.

1. **The method of measuring slow-phase eye movements**

In the VOR experiments, we recorded the volunteers’ eye movements by the small infrared camera which was fixed on the eyepatch. The recorded videos were analyzed using MATLAB R2017b. The center of the pupil was tracked and located. In the nystagmus, the velocity of eye movement (*EV*) can be written as follows:

$$EV=\frac{\theta}{t}$$

Where *θ* is the angle of eye rotation, *t* is the time.

As shown in the Figure S2, we constructed the simplified model for the eye movement. The eyeball was simplified to a circle whose center point O was the center of the eyeball. We assumed that the center of the pupil moved to the points, O_1_, O_2_, and O_3_ at different times of *t_1_*, *t_2_*, and *t_3_*, respectively. Besides, the point O_0_ represented that the center of the pupil was in middle position of the eye. We measured the X-coordinate value of points, O_0_, O_1_, O_2_, and O_3_, respectively by tracking and locating the pupil. The unit was pixel. When the center of the pupil moved from point O_1_ to O_2_, the transient velocity of eye movement denoted as *EV_1_* could be written as follows：

$$EV_{1}=\frac{\theta_{2}}{t_{2}-t_{1}}$$

$$\theta_{2}=arcsin\left( \frac{X_{2}-X_{0}}{R} \right)-arcsin\left( \frac{X_{1}-X_{0}}{R} \right)$$

Where *θ_2_* was the angle of the center of the pupil moving from point O_1_ to O_2_, *X_0_* was the value of X-coordinate pixels when the center of the pupil was in middle position, *X_1_* and *X_2_* were the value of X-coordinate pixels when the center of the pupil was in the position of point O_1_ and O_2_, respectively, and *R* was the pixel value of the rotational radius of the eyeball.

The volunteer wearing the eye patch sat on the rotatable chair before the experiment began. They were told to move their eyes to the far left and the far right. Then, we measured the X-coordinate value of the center of the pupil when the volunteers’ eyes were in position of the far left and the far right, and calculated their mean that was *X_0_*. Besides, we obtained the actual radius of the pupil by measuring pixel size of the pupil’s radius, and the proportional relationship between the actual size of the reference object and its pixel size in the image. Then, we calculated the pixel size of the rotational radius of the eyeball in the image according to the proportional relationship between the radius of the pupil and the rotational radius of the eyeball. Considering the small individual differences in the radius of eyeballs, the rotational radius of the volunteers’ eye was set to 13.5 mm (Park et al., 1933; Lee et al., 2009). The horizontal nystagmus SPV was calculated by this method. Besides, the calculation method of the vertical nystagmus SPV was similar to that of the horizontal nystagmus SPV.

1. **Additional VOR experiment to determine the time of the volunteer’s SPV becoming stable**

In order to determine whether the experimental time of 7 seconds can make the volunteer’s SPV reach a steady state, we performed an additional VOR experiment for another volunteer whose head was rotated anticlockwise with the constant angular acceleration of 15°/s^2^ for 14s (The rotational stimulus was shown in Figure S3) when the volunteer’s head was in the normal position. The volunteer’s nystagmus and SPV changing with time were shown in the Figure S4 and Figure S5, respectively. The volunteer’s SPV increased within 5 s after the head experienced the change in constant angular acceleration of 15°/s^2^, and then reached an approximate steady state. The volunteer’s SPV decreased after the head sensed the constant rotational velocity.

1. **The consequences of exciting/inhibiting signals on the VOR**

As Eggers et al. (2019) described in their study, Figure S6A shows that excitation of the left horizontal (LH) SCC induces rightward slow phases mainly as a result of strong activation of right lateral rectus (LR) and left medial rectus (MR). Figure S6B shows that excitation of the left anterior (LA) SCC induces upward/clockwise (from volunteer’s perspective) slow phase because of the combined action of the right inferior oblique (IO) and superior rectus (SR) and the left superior oblique (SO) and SR (i.e. the corresponding extraocular muscles are activated). Figure S6C shows that excitation of the left posterior (LP) SCC induces downward/clockwise (from volunteer’s perspective) slow phase as a result of the combined action of the right IO and inferior rectus (IR) and the left SO and IR. Figure S6D shows that combined equal excitation of both the left anterior (LA) and right anterior (RA) SCCs activates bilateral SR and oblique muscles and causes purely upward slow phases since the torsional components from each SCC cancel each other. Figure S6E shows that combined equal excitation of left anterior (LA) and left posterior (LP) SCCs excites muscle activity that is the sum of each SCC’s individual effect; upward and downward pulls cancel, which results in a purely torsional nystagmus. Figure S6F shows that combined equal excitation of all three left SCCs induces a right-clockwise (from volunteer’s perspective) slow phase, the expected result of summing activity for each individual SCC.

In the results of this study, when the forward-leaning angle of the head was less than 30°, LA and LP SCCs were excited, similar to the situation in the Figure S6E. However, when the volunteer’s head was tilted 0° to the forward, there was no significant torsional nystagmus in the VOR experiment. The sum of LA and LP SCCs’ cupula shear strain at the crista surface didn’t change significantly when the forward-leaning angle of the head was less than 30°. This might be the reason why volunteers’ the torsional nystagmus was not significant when the forward-leaning angle of the head was less than 30°. Besides, RA and RP SCCs were inhibited when the forward -leaning angle of the head was less than 30°. Then, the corresponding extraocular muscles were not activated by RA and RP SCCs, which wouldn’t induce the corresponding nystagmus. When the forward-leaning angle of the head was 30°, LA SCC was excited, similar to the situation in the Figure S6B. LP and RA SCCs were not excited, and RP SCC was inhibited, which wouldn’t activate the corresponding extraocular muscles and induce the corresponding nystagmus. When the forward-leaning angle of the head was greater than 30°, LA and RA SCCs were excited, similar to the situation in the Figure S6D. LA and RA SCCs’ cupula shear strain at the crista surface had a similar tendency of variation, and the magnitude of them was approximately the same (see Figure 3C and 3D). Then, the torsional nystagmus of volunteers was not significant in the VOR experiments since the torsional components from each SCC cancel each other. Besides, RP and LP SCCs were inhibited which wouldn’t activate the corresponding extraocular muscles. Thus, RP and LP SCCs could not induce the corresponding nystagmus.

1. **The effects of different rotation radius in the model and VOR experiment**

In the VOR experiments, the rotation axis of the chair always passes through point N0, and is perpendicular to the XY plane (that is in the positive direction of Z, see Figure S7). Thus, the heads of the volunteers were always rotated around the rotation axis of the chair under different head positions. The center of rotation was N0 during VOR experiments. The volunteers tilted their body to achieve different head orientations, which caused a change in the distance between the head and the rotation axis of the chair. As the left-leaning angle of the head increases, the rotation radius of the SCCs increases. The rotation radii of the bilateral SCCs were approximately 10 cm when the volunteer’s head tilted 90° to the left. In the normal head position, the rotation radii of the bilateral SCCs were approximately 3 cm. Then, we compared the volunteers’ SPV under normal head position when the rotation radius was 3 cm and 10 cm, respectively. In the VOR experiments, the rotation radius of the volunteers’ left SCCs was approximately 3 cm and 10 cm, respectively (see Figure S8). The chair rotated clockwise for 7 s with a constant angular acceleration of 30°/s^2^, and an initial rotational velocity of 0.

For the numerical model of the bilateral SCCs, the rotation centre is P0 for all models. The rotation radii of the bilateral SCCs also changed with the different head positions. Then, we compared the maximal cupula shear strain under normal head position when the left SCCs’ rotation radius was 3 cm and 10 cm respectively (i.e., right SCCs’ rotation radius was 3 cm and 4 cm respectively). In the numerical model, the rotation radius of the left SCCs was 3 cm and 10 cm, respectively (see Figure S8), corresponding to the rotation radius of the volunteers’ left SCCs of 3 cm and 10 cm in the VOR experiment. Both the left and right SCCs were loaded with a clockwise angular acceleration of 30°/s^2^ for 30 s, and an initial rotational velocity of 0.

Figure S9 shows three volunteers’ SPV changing with time under normal head position when the rotation radius was 3 cm and 10 cm, respectively. The volunteer’s SPV became approximately stable after 5 s. Then, we calculated the mean of SPV during 5-7 s (see Table S1). Besides, Figure S10 shows the cupula shear strain in the left and right SCCs changing with time under a normal head position when the rotation radius of left SCCs was 3 cm and 10 cm, respectively.

In addition, considering that it was difficult for the rotation radius of the volunteer’s head to reach 3 cm when the head was tilted 90° to the left, we could only investigate the cupula shear strain in the numerical model of bilateral SCCs when the rotation radius was 3 cm and 10 cm, respectively, under the condition of the head tilted 90° to the left. As shown in the Figure S11, the rotation axes of bilateral SCCs are Z2 and Z1, respectively, corresponding to rotation radii of 3 cm and 10 cm, respectively. Both the left and right SCCs were loaded with a clockwise angular acceleration of 30°/s^2^ for 30 s, and an initial rotational velocity of 0. Figure S12 shows the maximal cupula shear strain in the left and right SCCs changing with time under the condition of the head tilted 90° to the left when the rotation radius was 3 cm and 10 cm, respectively.

The results of the numerical simulation and VOR experiments above indicate that the changes in the rotation radius within 10 cm will not have a significant impact on the maximal cupula shear strain and volunteers’ SPV under a constant angular acceleration of 30°/s^2^ at the same head position. Although the rotation radius of SCCs in the numerical model and the VOR experiment may not be completely consistent in this study, it will not have a significant effect on the research results.

1. **Cupula time constant**

The cupula time constant reflects the geometry of SCCs and the cupula, as well as the physical properties of the cupula and endolymph (Rabbitt et al., 2004). The cupula time constant in the numerical model can be determined by fitting a curve with exponential decay to $y_{c}(t)$ (D. Obrist et al., 2010).

$$y_{c}(t)\sim e^{-t/T_{c}}$$

*T_c_* represent the cupula time constant that is the time over which the cupula response to the step of rotation decays to 1/e of its initial value (Raphan et al., 1979; Cohen et al., 1992, 2004; Dai et al., 1999; Rabbitt et al., 2004; D. Obrist et al., 2010). We calculated the cupula time constant in the numerical model of SCCs based on the equation fitting the curve of cupula shear strain once the rotational velocity became constant (see Figure S13). The return phase of the cupula is governed by the cupula time constant (Van Buskirk et al., 1976).

1. **Endolymphatic pressure distribution under different forward-leaning head positions**

The figures depicting the endolymphatic pressure distribution in SCCs with the more detailed legends were shown in Figure S14 to S25.

1. **Comparison on the viscous, inertial, and convective terms in the regions of narrow SCCs**

When employing the rotating SCCs as a reference frame, we compared viscous, inertial, and convective terms within the fluid domain. The maximum magnitudes of inertial and convective terms were comparable in the regions of narrow SCCs, while the convective term could be considered negligible (see Figure S26). At the initial moment, there was an increase in inertial forces in the regions of narrow SCCs. As time increased, the inertial force decreased to a negligible level, and the viscous force increased, while the convective forces could be considered negligible in the time domain. This phenomenon might arise due to the deformation of the cupula caused by the pressure gradient, leading to relative flow in the regions of narrow SCCs. At the initial moment, there was a noticeable relative flow velocity of endolymph within the narrow canals (see Figure S27A). As the displacements of the cupulae gradually increased to a stable level, the relative flow velocity of endolymph within the canal gradually decreased to a negligible level (see Figure S27B). These results we obtained were similar to those in the study by Goyen et al. (2019).

1. **References**

Boselli, F., Obrist, D., Kleiser, L. (2013). Vortical flow in the utricle and the ampulla: a computational study on the fuid dynamics of the vestibular system. Biomech. Model. Mechanobiol. 12, 335-348.

Eggers, S.D.Z., Bisdorff, A., Brevern, M.V., Zee, D.S., Newman-Toker, D.E. (2019). Classification of vestibular signs and examination techniques: nystagmus and nystagmus-like movements: consensus document of the committee for the international classification of vestibular disorders of the bárány society. J. Vestib. Res. 29, 1-31.

Goyens, J., Pourquie, M., Poelma, C., Westerweel, J. (2019). Asymmetric cupula displacement due to endolymph vortex in the human semicircular canal. Biomech. Model. Mechanobiol. 18, 1577-1590.

Ifediba, M.A., Rajguru, S.M., Hullar, T.E., Rabbitt, R.D. (2007). The role of 3-canal biomechanics in angular motion transduction by the human vestibular labyrinth. Ann. Biomed. Eng. 35, 1247-1263.

Kassemi, M., Deserranno, D., Oas, J.G. (2005). Fluid–structural interactions in the inner ear. Comput. Struct. 83, 181-189.

Lee, E.C., Park, K.R. (2009). A robust eye gaze tracking method based on a virtual eyeball model. Mach. Vis. Appl. 20, 319-337.

Obrist, D., Hegemann, S., Kronenberg, D., Häuselmann, O., Rösgen, T. (2010). In vitro model of a semicircular canal: design and validation of the model and its use for the study of canalithiasis. J. Biomech. 43, 1208-1214.

Park, R.S., Park, G.E. (1933). The center of ocular rotation in the horizontal plane. Am. J. Physiol. Leg. Content. 104, 545-552.

Rabbitt, R.D., Damiano, E.R., Grant, W.J. (2004). In: Highstein, S.M., Fay, R.R. (Eds.). The Vestibular System. Springer, New York, USA, pp. 153–201.

Rajguru, S.M., Ifediba, M.A., Rabbitt, R.D. (2004). Three-dimensional biomechanical model of benign paroxysmal positional vertigo. Ann. Biomed. Eng. 32, 831-846.

Selva, P., Oman, C.M., Stone, H.A. (2009). Mechanical properties and motion of the cupula of the human semicircular canal. J. Vestib. Res. 19, 95-110.

Squires, T.M., Weidman, M.S., Hain, T.C., Stone, H.A. (2004). A mathematical model for top-shelf vertigo: the role of sedimenting otoconia in bppv. J. Biomech. 37, 1137–1146.

# Supplementary Figures and Tables


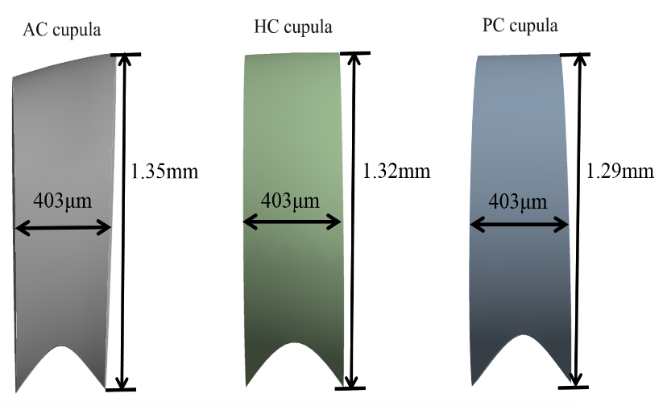


Figure S1. The geometries of three cupulae in SCCs


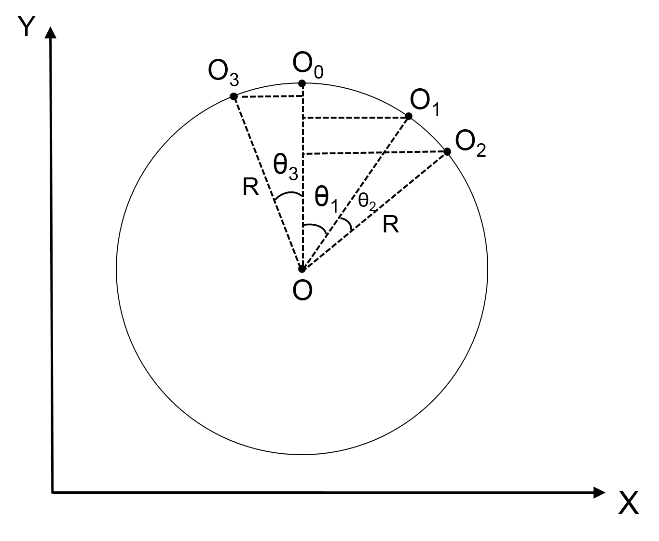


Figure S2. Simplified model for eye movement.


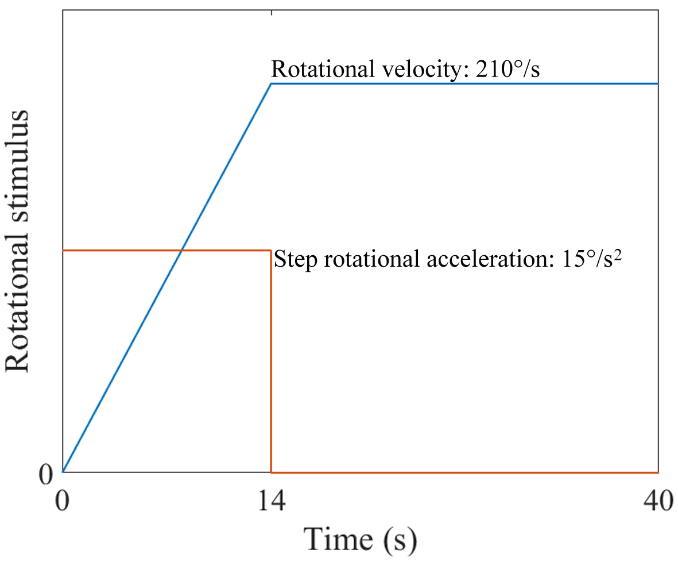


Figure S3. The rotational stimulus in the additional experiments


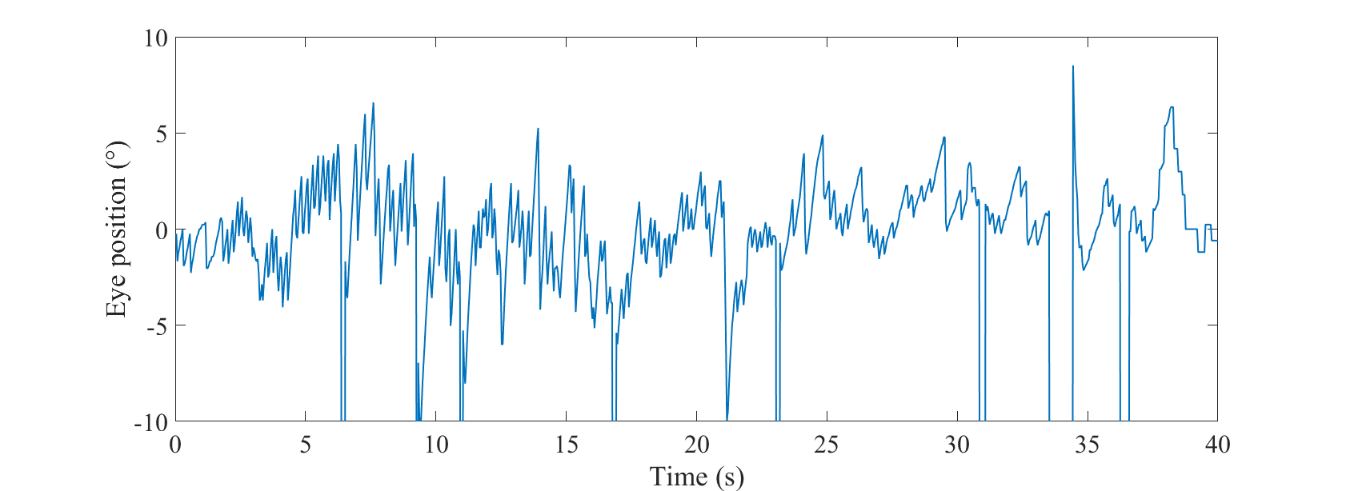


Figure S4. The horizontal nystagmus of a volunteer under normal head position. The interruption in the nystagmus trajectory represents the volunteer blinking eye.


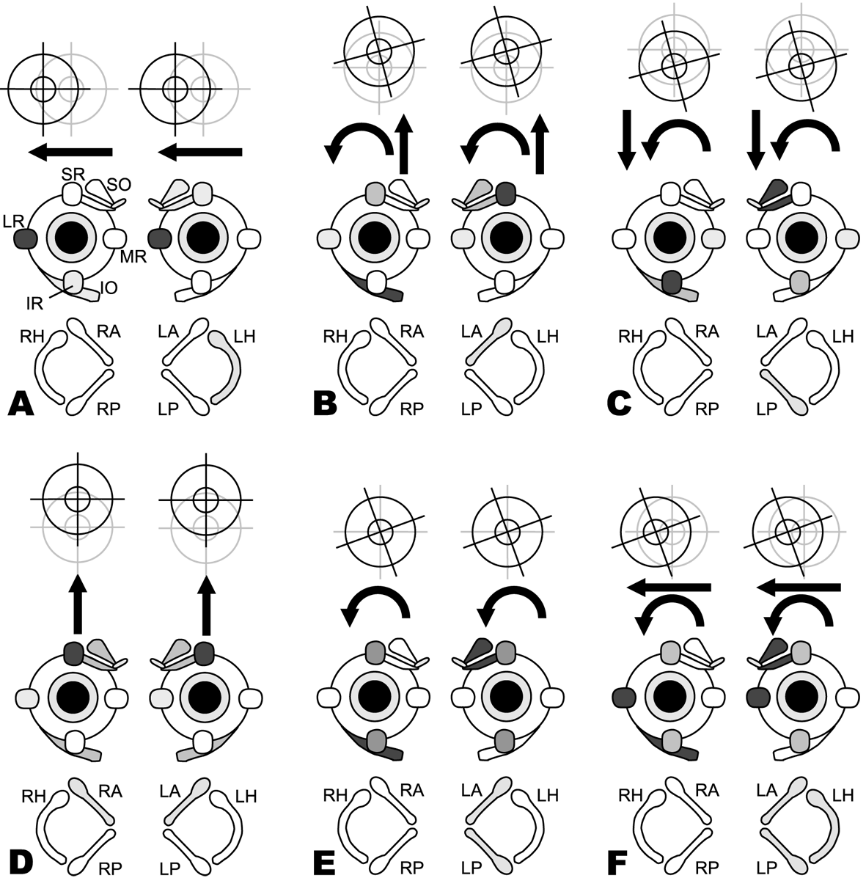


Figure S6. Nystagmus slow phases observed for excitation of individual SCCs (Eggers et al., 2019). In the bottom row of each panel (A through F), shading indicates the excited canals. In the second row, a diagram of the extraocular muscles depicts which muscles are activated (darker shading indicates stronger activation). In the top row, the resultant yaw, pitch, and/or roll eye movements are indicated.


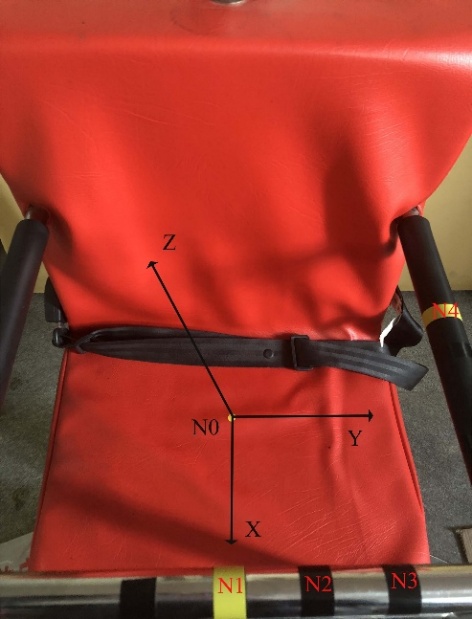


Figure S7. The rotatable chair. The rotation center is the point N0, and the rotation axis passes through the point N0, in the positive direction of Z.


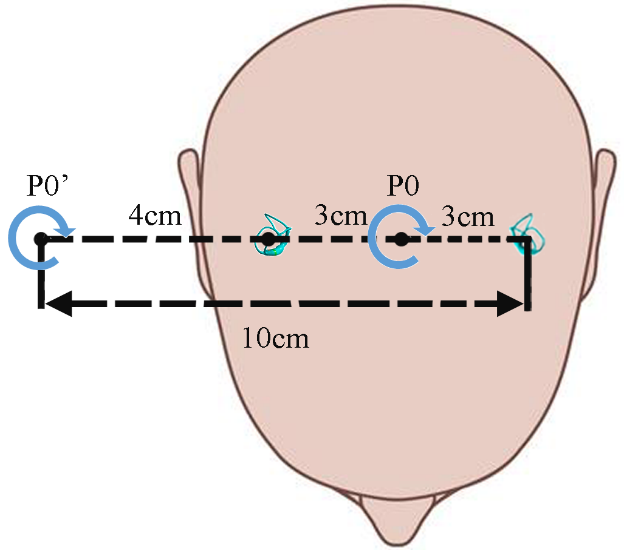


Figure S8. Different rotation radii for bilateral SCCs under normal head position. The rotation centre is P0 when the rotation radius of bilateral SCCs is 3 cm. The rotation center is P0’ when the rotation radius of left SCCs is 10 cm, and the rotation radius of right SCCs is 4 cm.


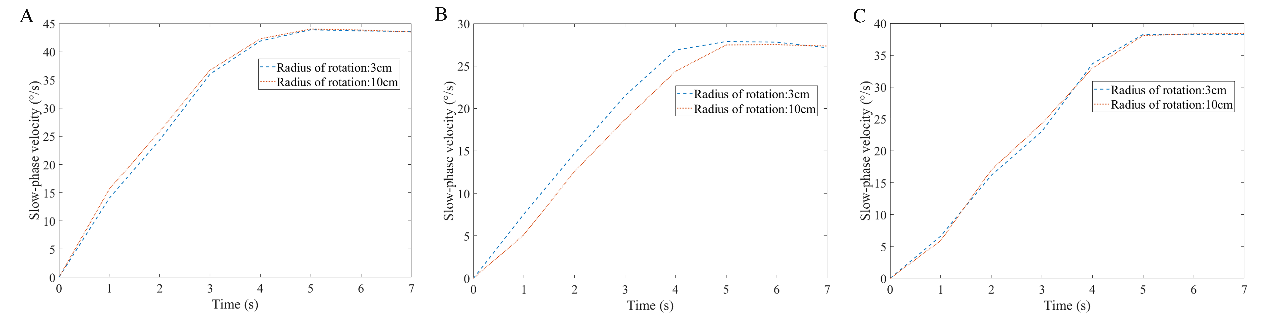


Figure S9. Three volunteers’ SPV changing with time under normal head position (i.e., head tilted 0° to the left) when the rotation radius was 3 cm and 10 cm respectively. (A) First volunteer’s SPV changing with time when the rotation radius was 3 cm and 10 cm respectively. (B) Second volunteer’s SPV changing with time when the rotation radius was 3 cm and 10 cm respectively. (C) Third volunteer’s SPV changing with time when the rotation radius was 3 cm and 10 cm respectively.


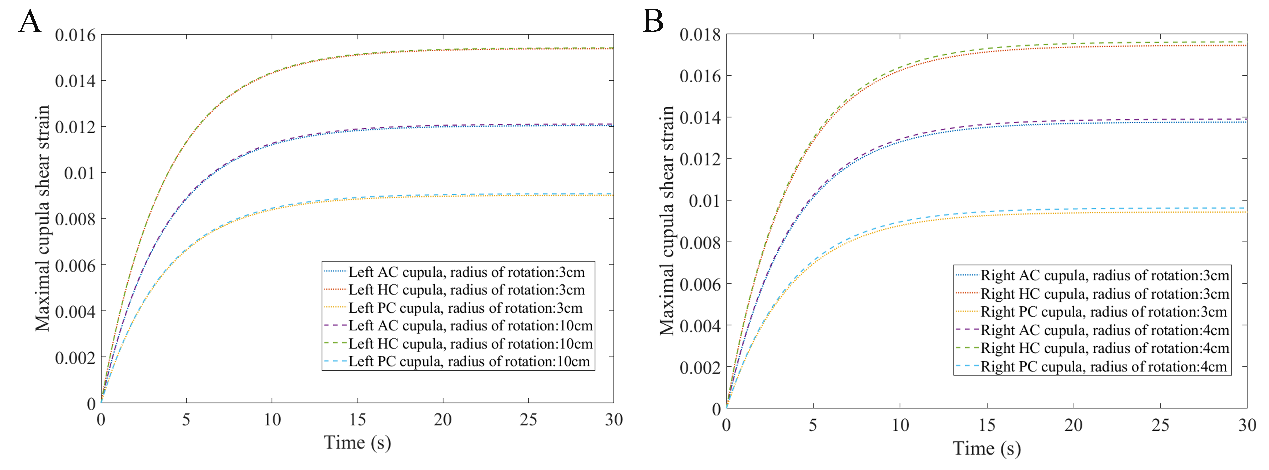


Figure S10. Maximal cupula shear strain in the normal head position under different rotation radii. (A) Maximal cupula shear strain in left SCCs when the rotation radius of left SCCs was 3 cm and 10 cm, respectively. (B) Maximal cupula shear strain in right SCCs when the rotation radius of right SCCs was 3 cm and 4 cm, respectively.


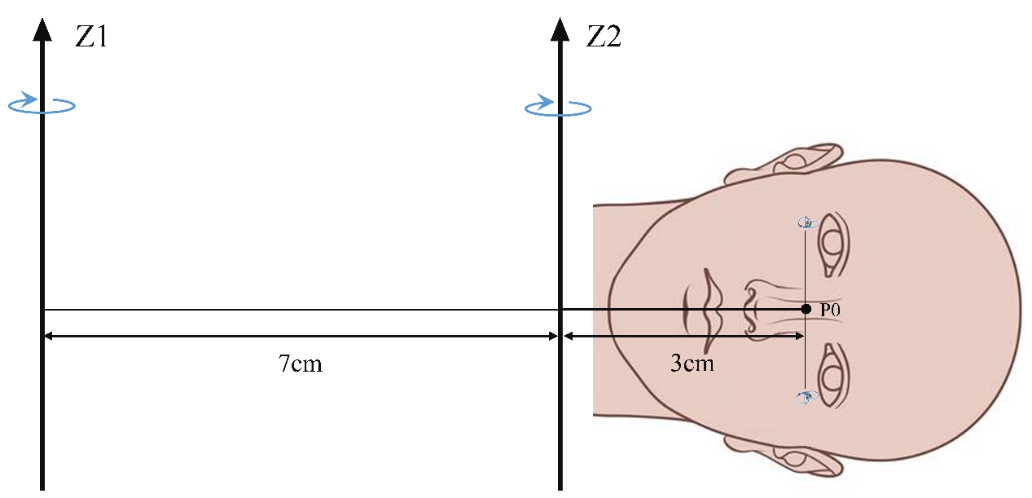


Figure S11. Different rotational radii for SCCs under the position of head tilted 90° to the left.


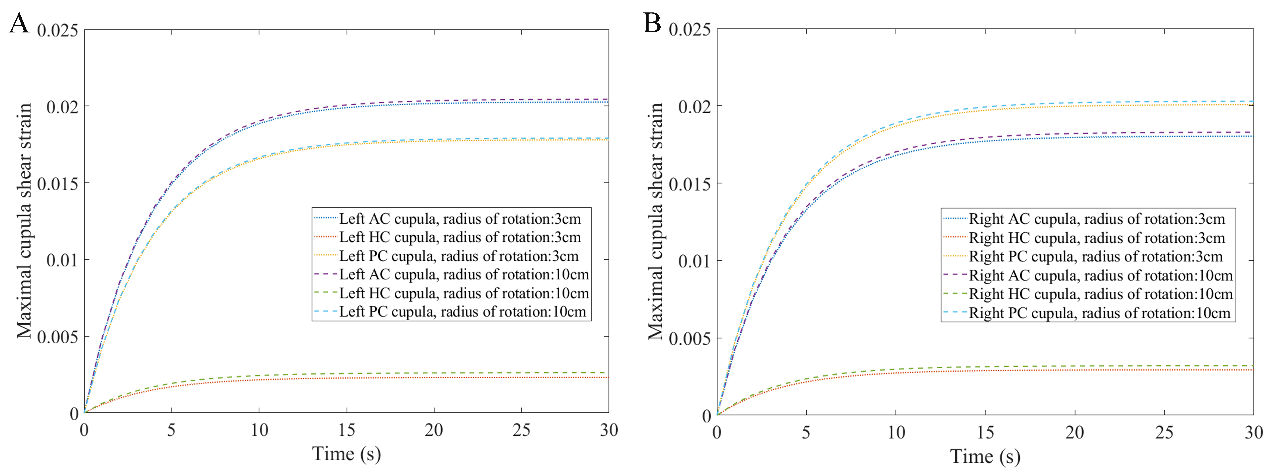


Figure S12. Maximal cupula shear strain with the head tilted 90° to the left under different radii of rotation. (A) Maximal cupula shear strain in left SCCs when the rotational radius of left SCCs was 3 cm and 10 cm, respectively. (B) Maximal cupula shear strain in right SCCs when the rotation radius of right SCCs was 3 cm and 10 cm, respectively.


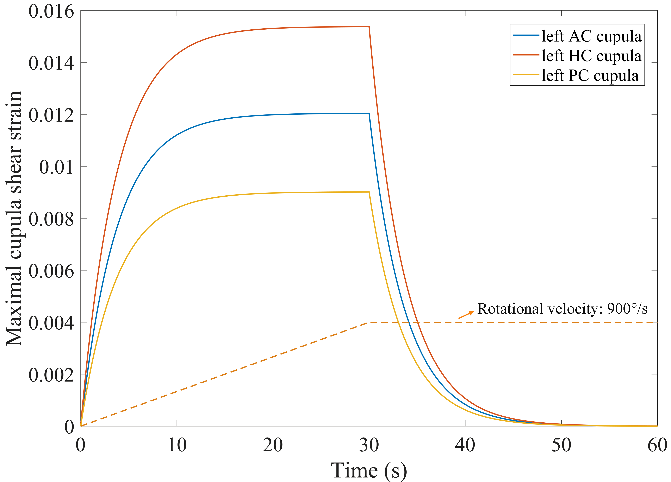


Figure S13. Maximal cupula shear strain in SCCs changing over time


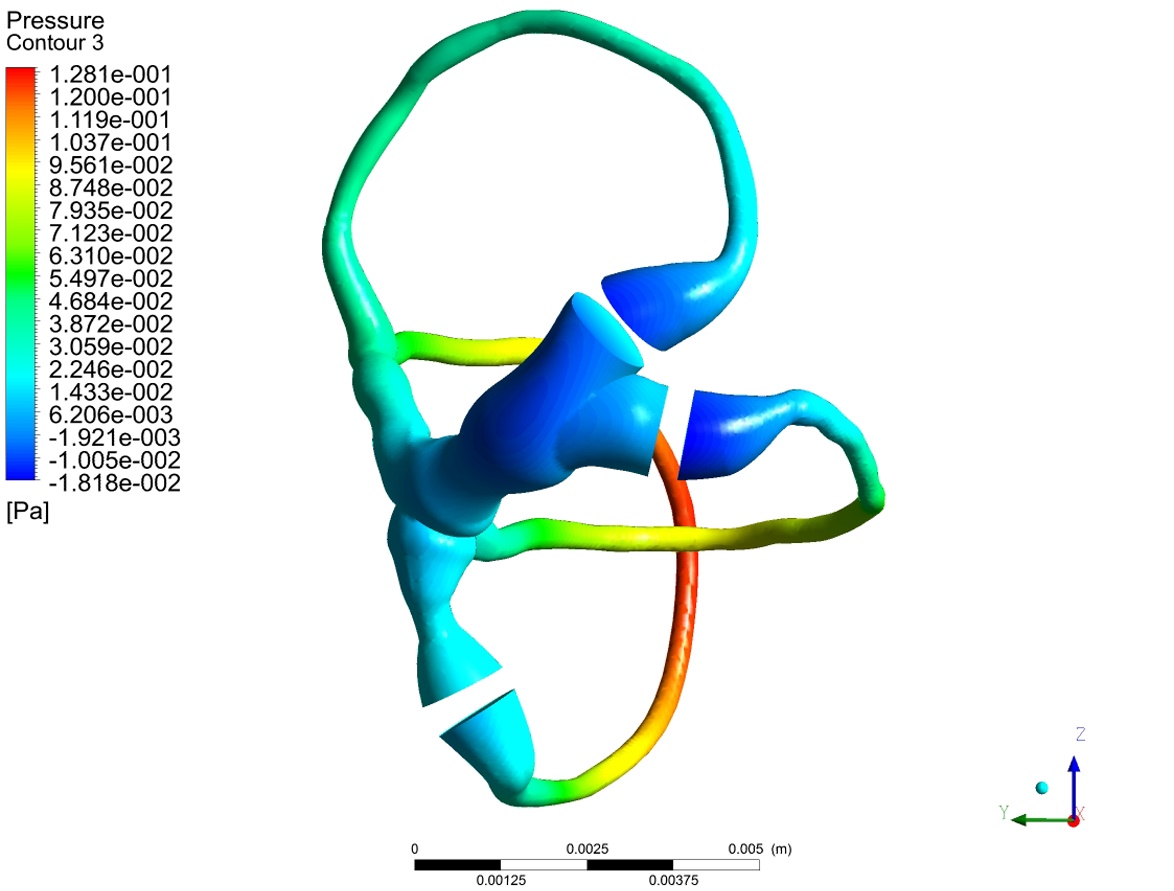


Figure S14 The endolymphatic pressure distribution in the left SCCs under head tilted forward 10°


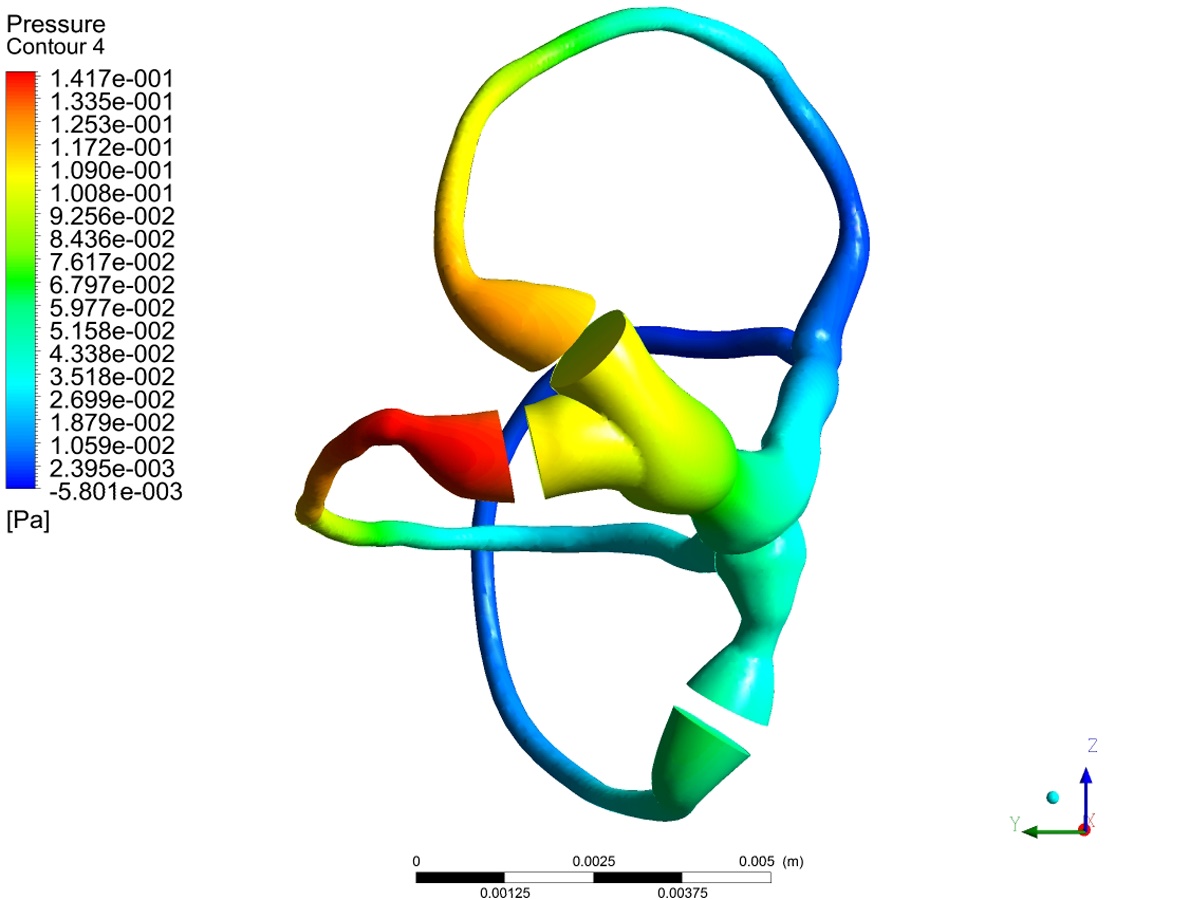


Figure S15 The endolymphatic pressure distribution in the right SCCs under head tilted forward 10°


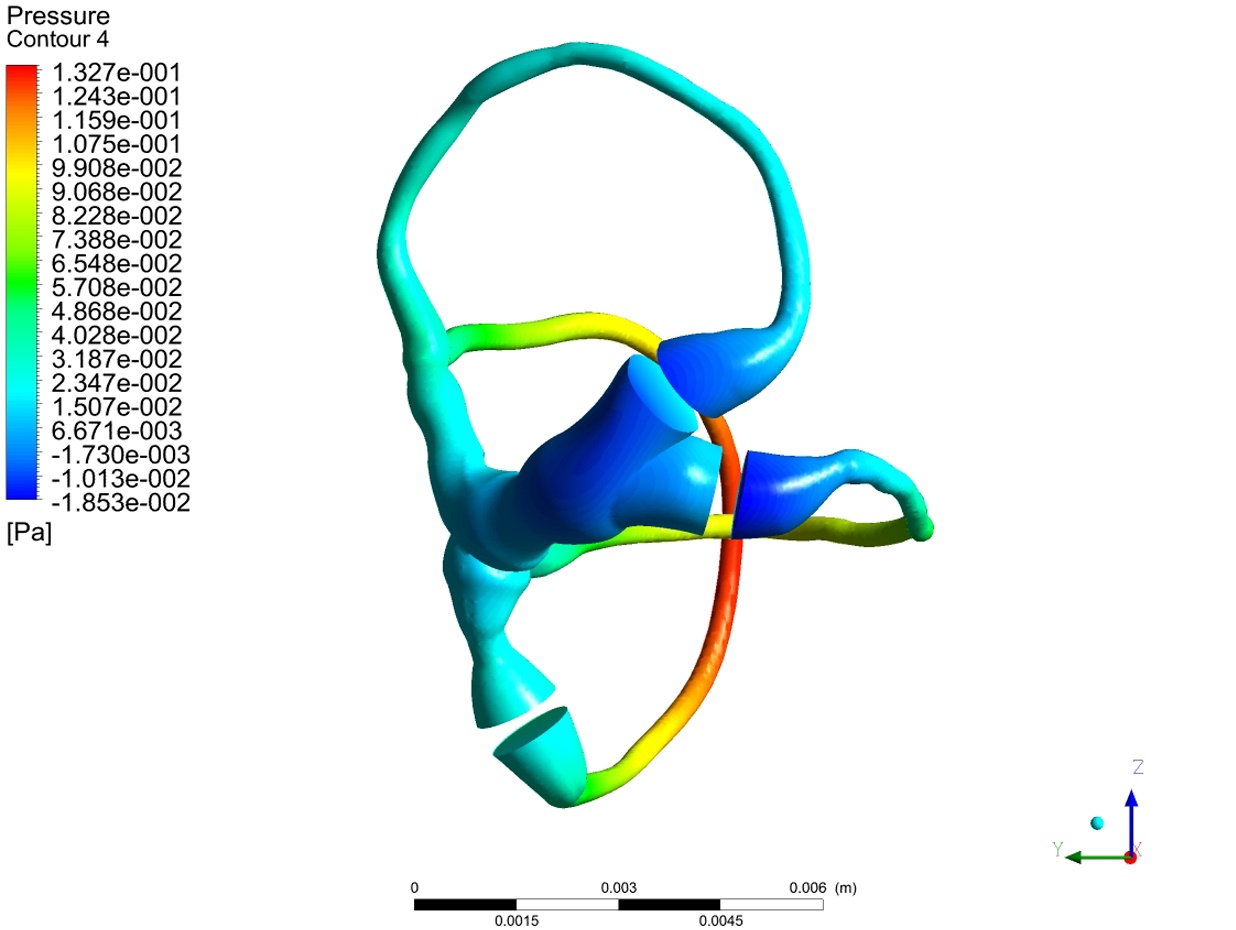


Figure S16 The endolymphatic pressure distribution in the left SCCs under head tilted forward 20°


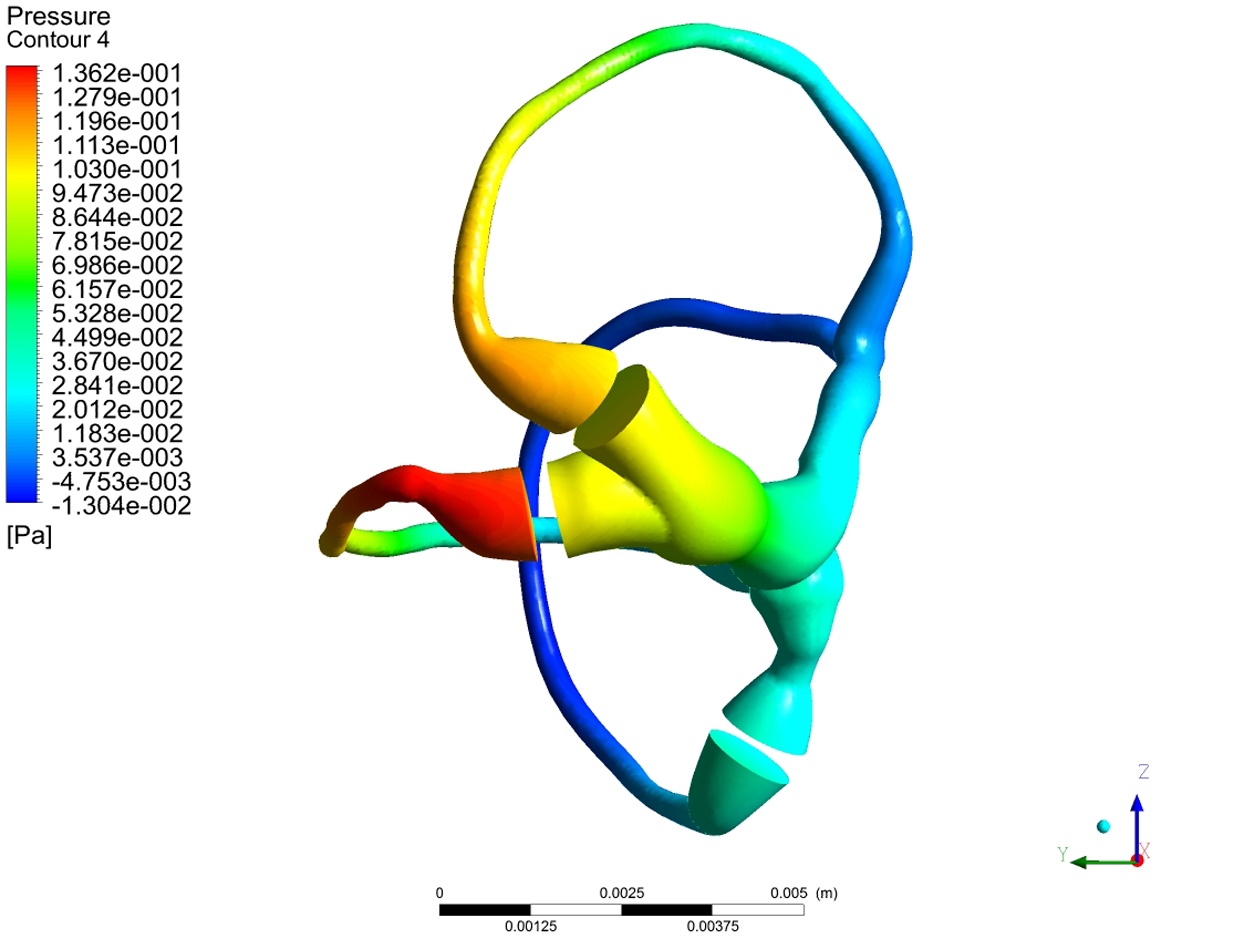


Figure S17 The endolymphatic pressure distribution in the right SCCs under head tilted forward 20°


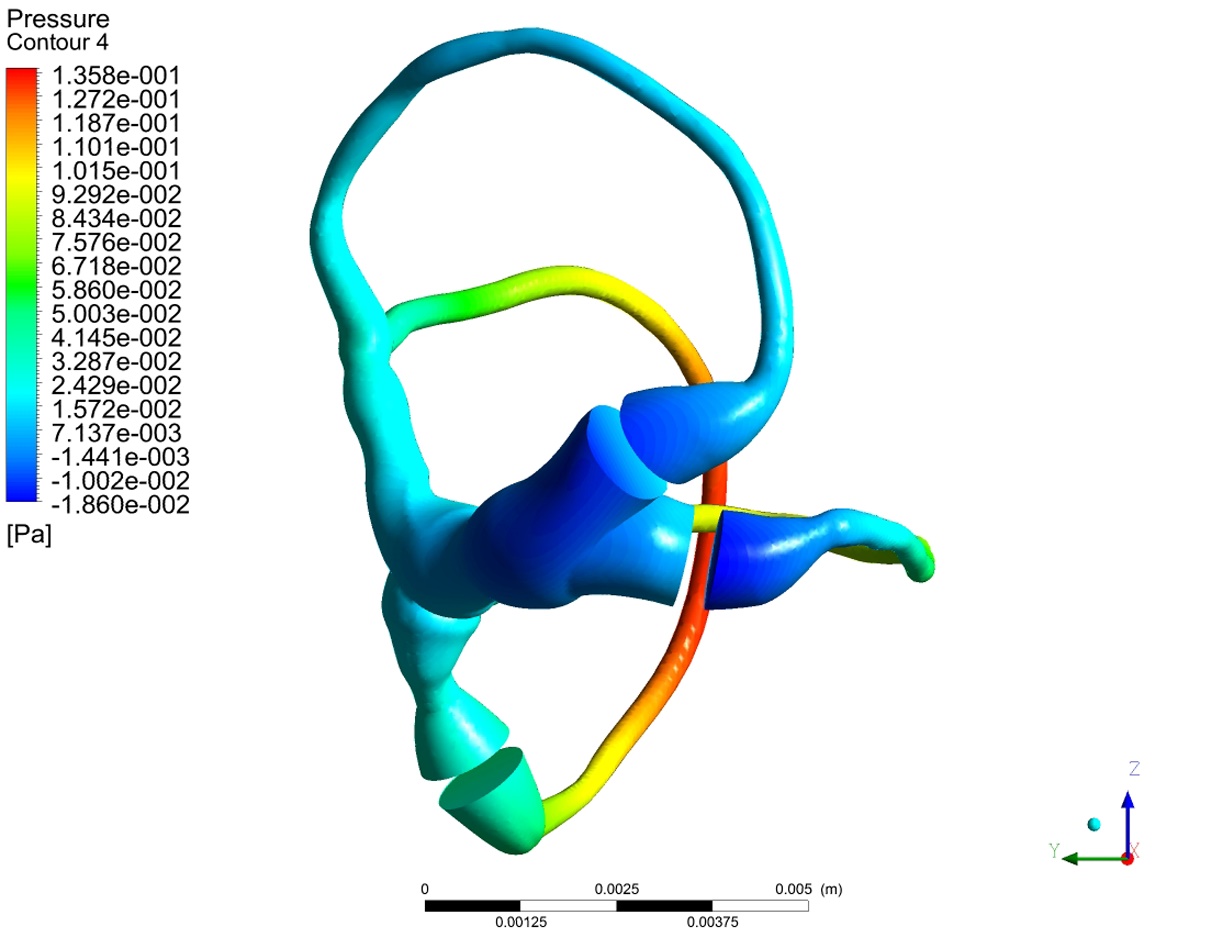


Figure S18 The endolymphatic pressure distribution in the left SCCs under head tilted forward 30°


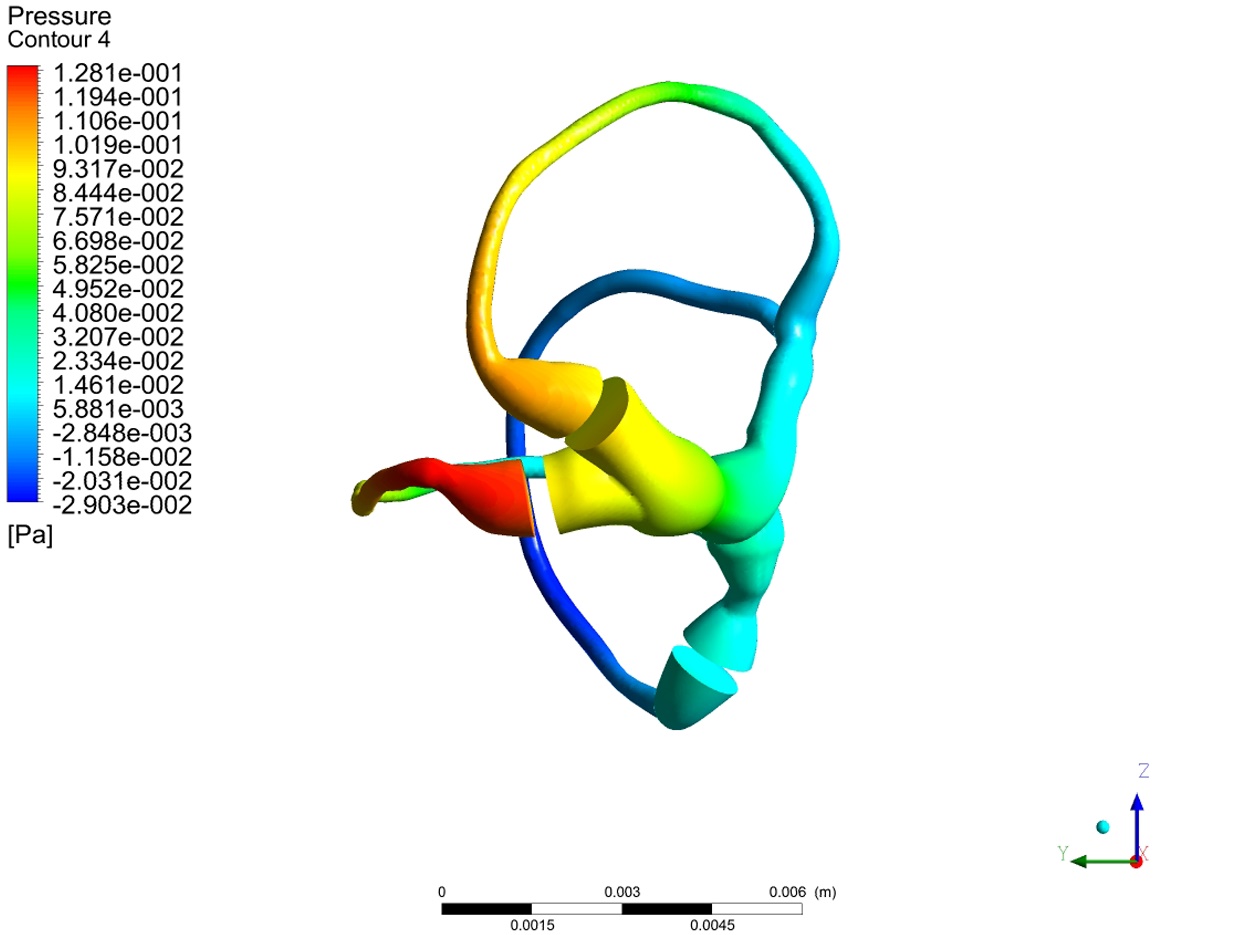


Figure S19 The endolymphatic pressure distribution in the right SCCs under head tilted forward 30°


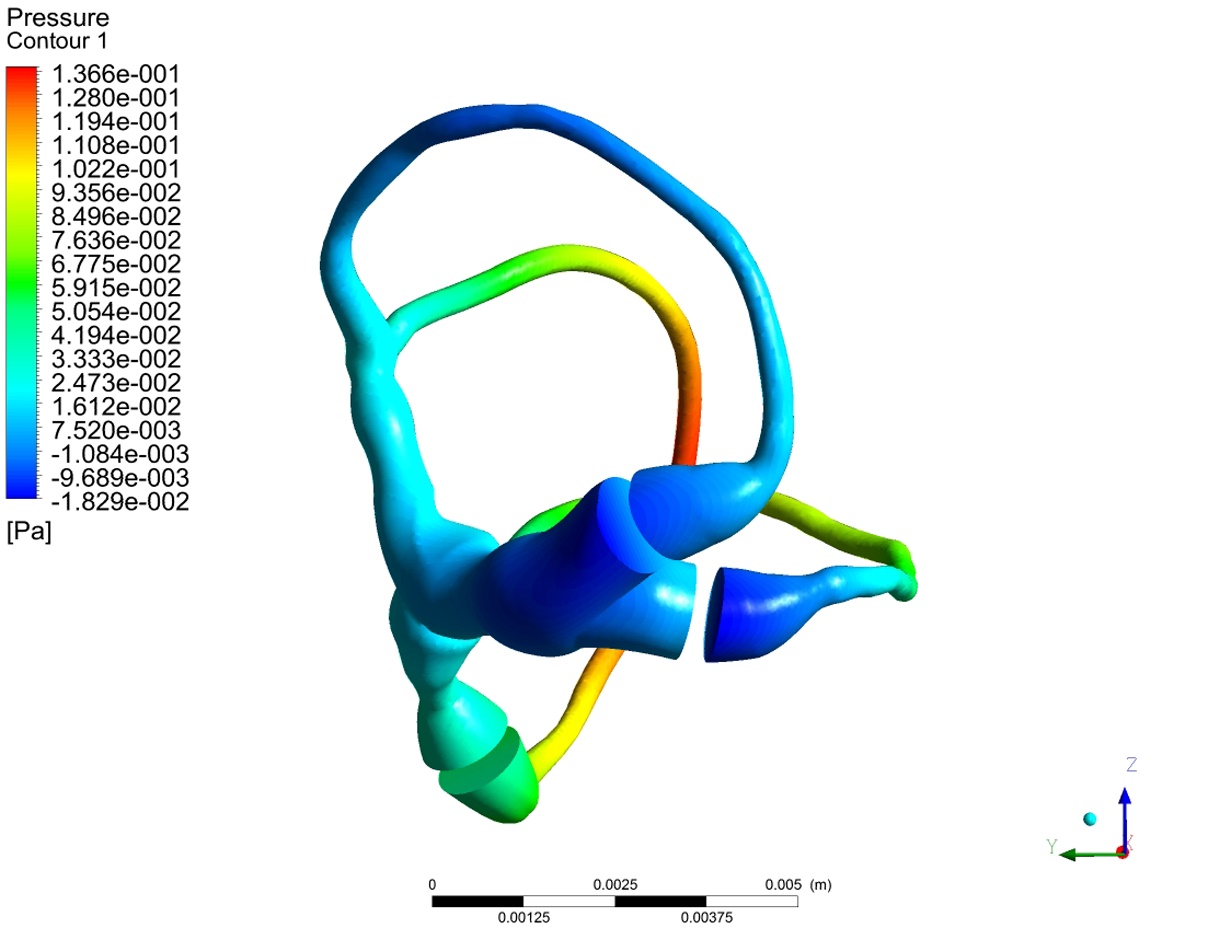


Figure S20 The endolymphatic pressure distribution in the left SCCs under head tilted forward 40°


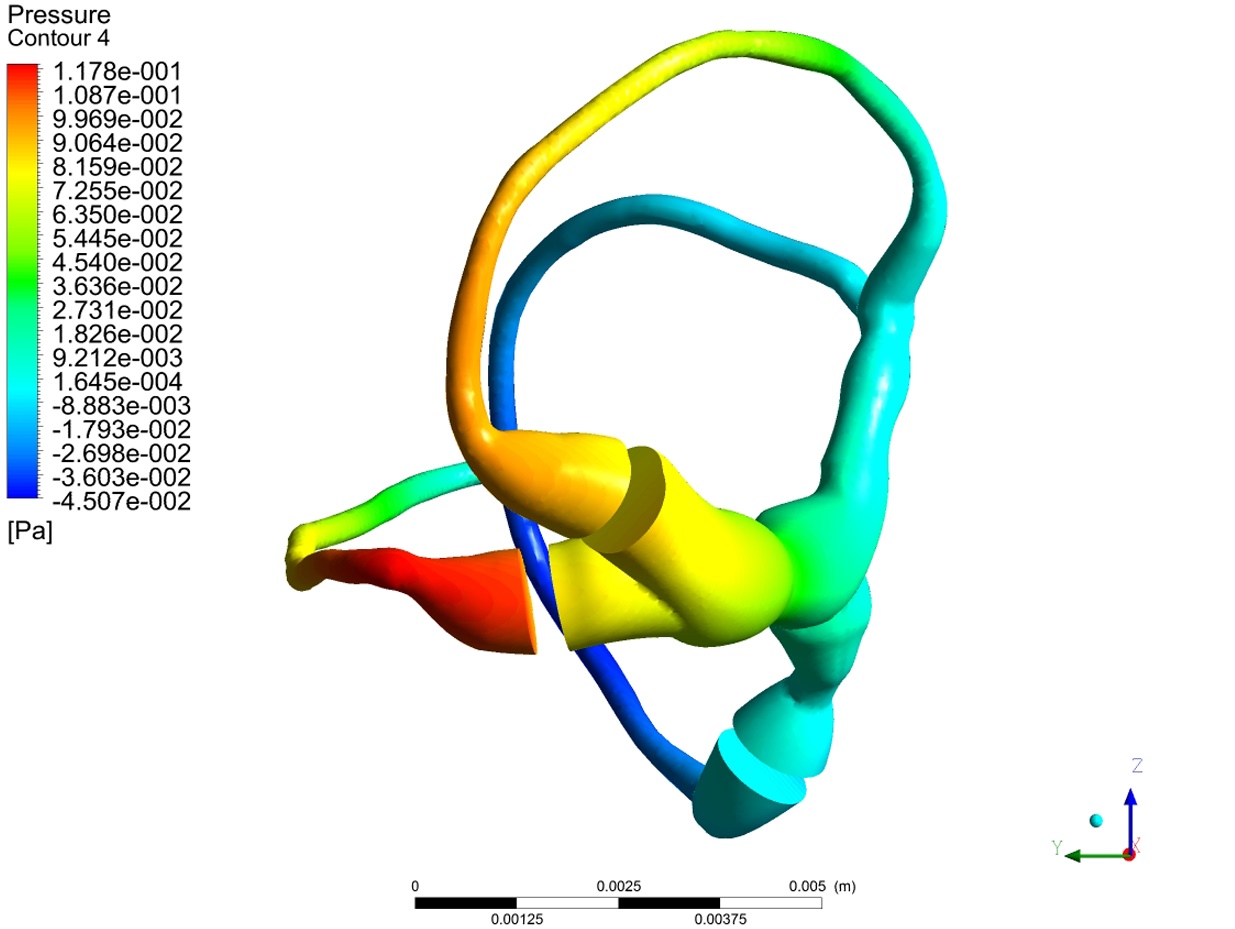


Figure S21 The endolymphatic pressure distribution in the right SCCs under head tilted forward 40°


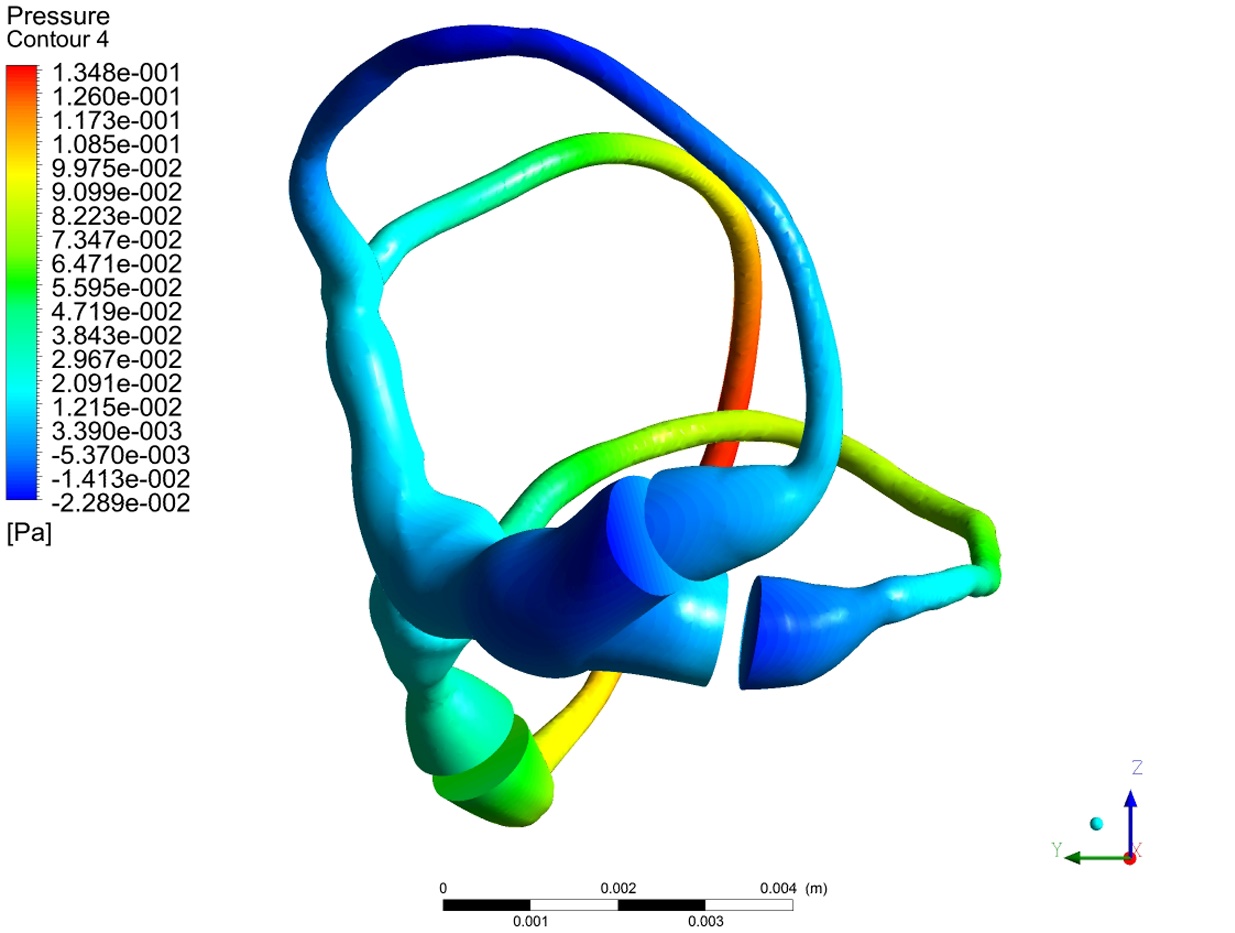


Figure S22 The endolymphatic pressure distribution in the left SCCs under head tilted forward 50°


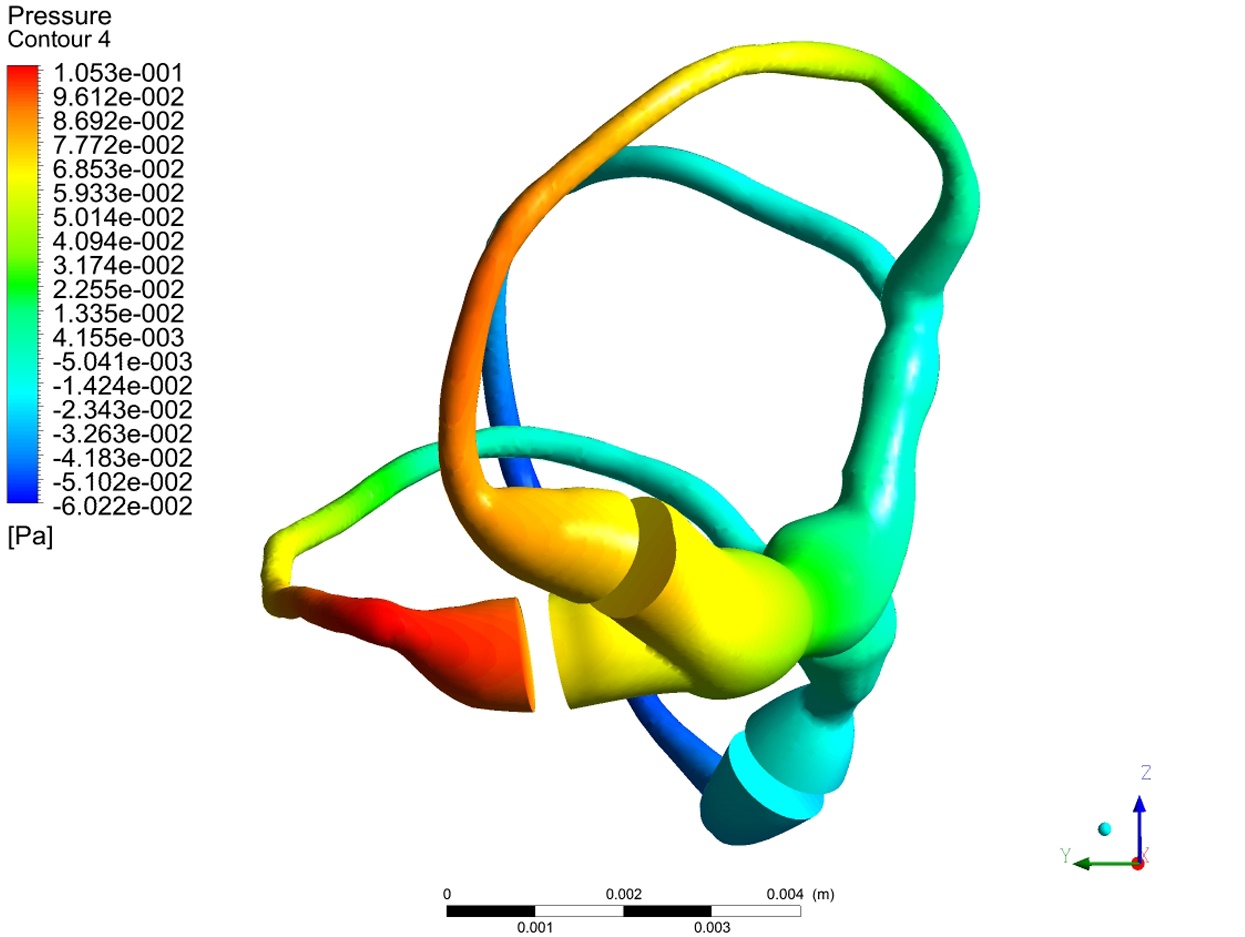


Figure S23 The endolymphatic pressure distribution in the right SCCs under head tilted forward 50°


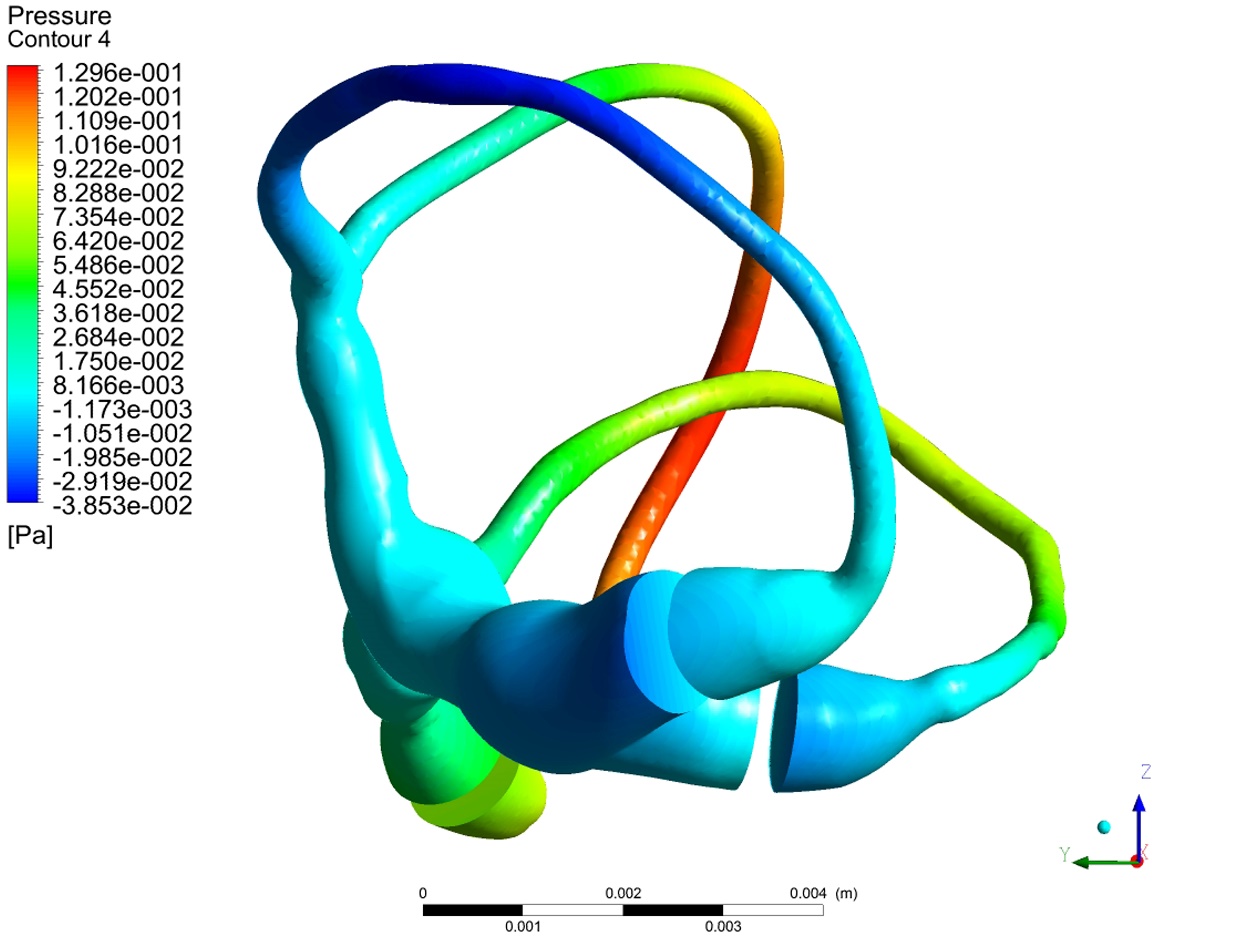


Figure S24 The endolymphatic pressure distribution in the left SCCs under head tilted forward 60°


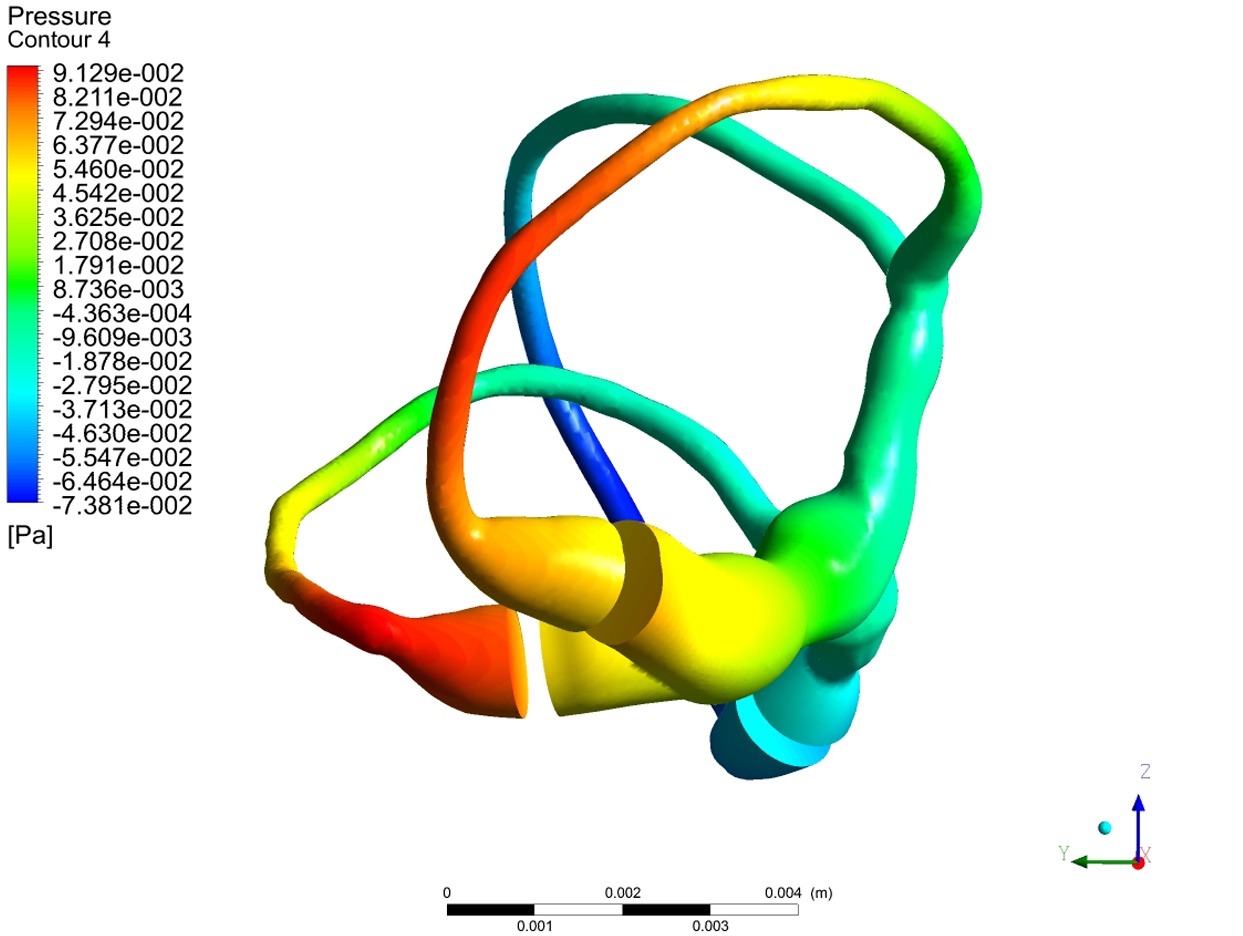


Figure S25 The endolymphatic pressure distribution in the right SCCs under head tilted forward 60°


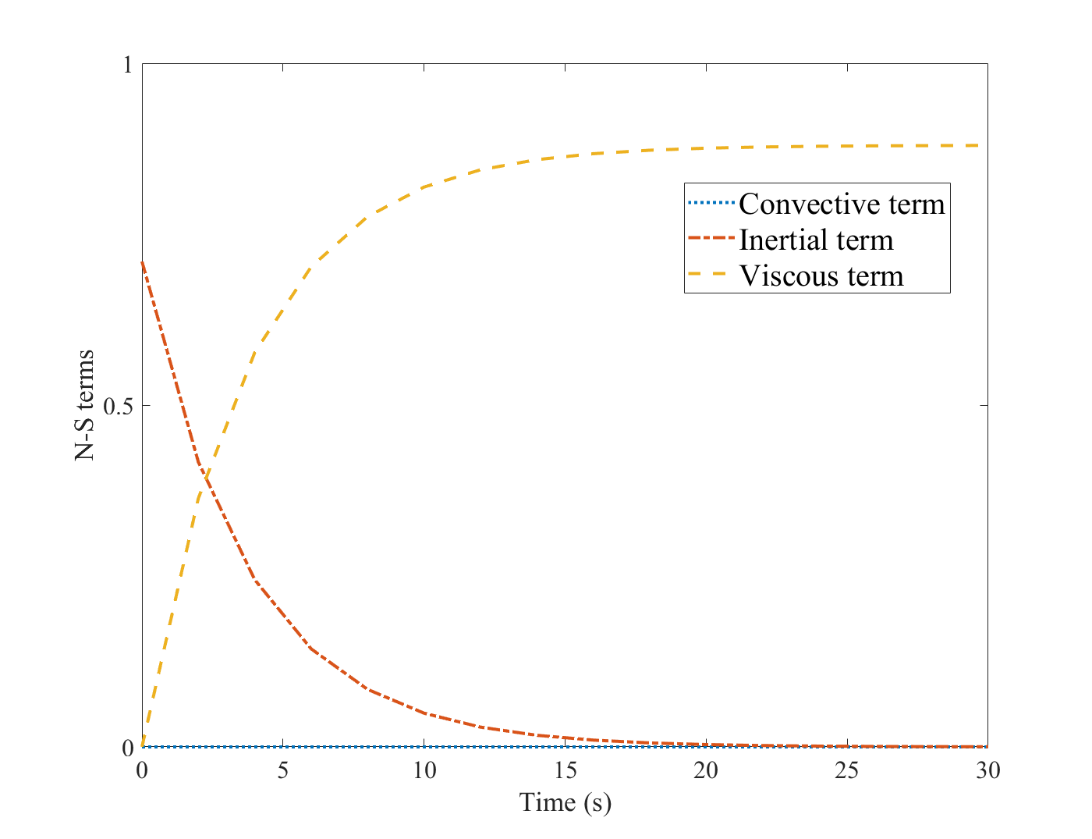


Figure S26 Comparison on the average scales of the convection term, inertia term, and viscosity term in the fluid regions of narrow SCCs


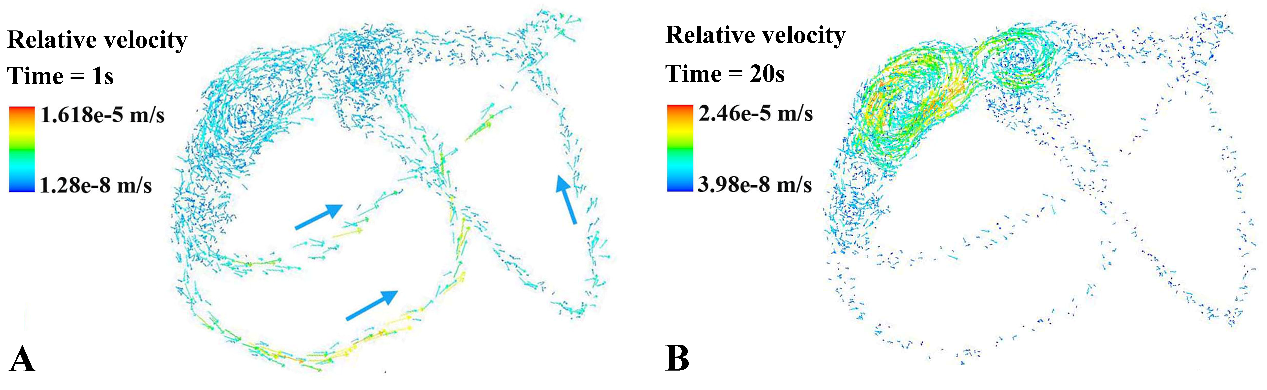


Figure S27 (A) Relative velocity of the endolymph at 1 s. (B) Relative velocity of the endolymph at 20 s

Table S1 Three volunteers’ SPV under normal head position with different rotation radius of 3 cm and 10 cm. The unit of SPV is °/s.

|  |  | SPV: mean (standard deviation) | |
| --- | --- | --- | --- |
|  |  | Rotation radius | |
|  |  | 3 cm | 10 cm |
| First volunteer | | 43.73(0.13) | 43.78(0.21) |
| Second volunteer | | 27.61(0.33) | 27.44(0.07) |
| Third volunteer | | 38.26(0.02) | 38.28(0.13) |
